# SMART: spatial transcriptomics deconvolution using marker-gene-assisted topic model

**DOI:** 10.1186/s13059-024-03441-1

**Published:** 2024-12-02

**Authors:** Chen Xi Yang, Don D. Sin, Raymond T. Ng

**Affiliations:** 1 Centre for Heart Lung Innovation, St. Paul’s Hospital, University of British Columbia, Vancouver, BC Canada; 2 Department of Bioinformatics, Faculty of Science, University of British Columbia, Vancouver, BC Canada; 3 Division of Respiratory Medicine, Department of Medicine, University of British Columbia, Vancouver, BC Canada; 4 Department of Computer Science, University of British Columbia, Vancouver, BC Canada

**Keywords:** Spatial transcriptomics, Deconvolution, Semi-supervised, Topic models

## Abstract

While spatial transcriptomics offer valuable insights into gene expression patterns within the spatial context of tissue, many technologies do not have a single-cell resolution. Here, we present SMART, a marker gene-assisted deconvolution method that simultaneously infers the cell type-specific gene expression profile and the cellular composition at each spot. Using multiple datasets, we show that SMART outperforms the existing methods in realistic settings. It also provides a two-stage approach to enhance its performance on cell subtypes. The covariate model of SMART enables the identification of cell type-specific differentially expressed genes across conditions, elucidating biological changes at a single-cell-type resolution.

**Supplementary Information:**

The online version contains supplementary material available at 10.1186/s13059-024-03441-1.

## Introduction

Spatial transcriptomics (ST) is a cutting-edge technology that enables scientists to measure gene expression patterns across different tissue regions with spatial information [1–3]. For most of the current ST platforms, the measured spots on a tissue sample do not demonstrate single-cell resolutions but contain a complex mixture of multiple cell types [4–6]. This hinders our understanding of the spatial organization of these cells and precludes the identification of cell type-specific transcriptomic signatures [7]. Understanding the cellular proportions and gene expression of specific cell types could better highlight cells and genes contributing to disease pathogenesis and identify therapeutic targets [8]. In silico deconvolution has been a promising approach to resolve the cellular composition at each measured spot. Most current ST deconvolution methods are reference-based, requiring a cell type-specific transcriptomic profile, usually generated from single-cell RNA-sequencing (scRNA-seq) experiments. With the reference profile, the cell type composition at each spot in the targeted ST dataset can be inferred. For example, RCTD [9] learns the cell type profile from the reference dataset using a probabilistic model and predicts the cell type composition of a spot with maximum likelihood estimation. SpatialDWLS [10] uses the scRNA-seq reference-derived signature to fit a dampened weighted least squares model to infer cell type composition. CARD [11], as an autoregressive-based deconvolution method, combines cell type-specific expression information learned from the scRNA-seq reference with correlation in cell type composition across the tissue spots. Cell2location [12] also borrows spatial information with a hierarchical Bayesian framework.

Despite the emerging number of scRNA-seq datasets, the desired reference profile may not be available for specific cell types or conditions [4]. The performance of these reference-based methods also highly depends on the quality, the sample processing techniques, and the data processing procedures of the reference profile. The inferred cell types are also limited to those available in the reference profile. Additionally, in some methods, the batch effects between the target ST dataset and the reference profile are not properly handled, resulting in inaccurate results [5]. Most importantly, by using a reference cell type-specific transcriptomic profile, it is assumed that the gene expression of each cell type is constant regardless of sample conditions such as disease status, ignoring the fact that there could be major differences in both the cell type composition and cell type-specific gene expression across different sample conditions.

In contrast, reference-free and marker-gene-assisted methods do not require a scRNA-seq reference but rely on a list of marker genes for each cell type. STdeconvolve [13], a Latent Dirichlet Allocation [14]-based reference-free method, decomposes the ST data into latent topics and simultaneously infers the topic-specific transcriptomic signature and topic compositions. Subsequently, by comparing the inferred topic-specific signature against known cell type marker genes with a gene set enrichment analysis (GSEA), each topic can be labeled with a cell type name. However, the estimated topics are usually redundant and cannot accurately capture cell populations with low abundance. Users may obtain multiple topics corresponding to an abundant cell type and no topics for rare cell types. The estimates are also highly variable between runs, even when using the same dataset. Compared to STdeconvolve, which uses marker genes after deconvolution, the marker-gene-assisted methods use marker genes during the deconvolution process. CARD offers a marker-gene-assisted version (CARDfree [11]) that takes a list of marker gene symbols as the input to infer the cell type composition. However, the inferred cell types can be difficult to interpret and need to be further labeled with a GSEA. Celloscope [15] is another Bayesian probabilistic model-based marker gene-driven approach that inflates the prior means of the marker genes.

**Fig. 1:**
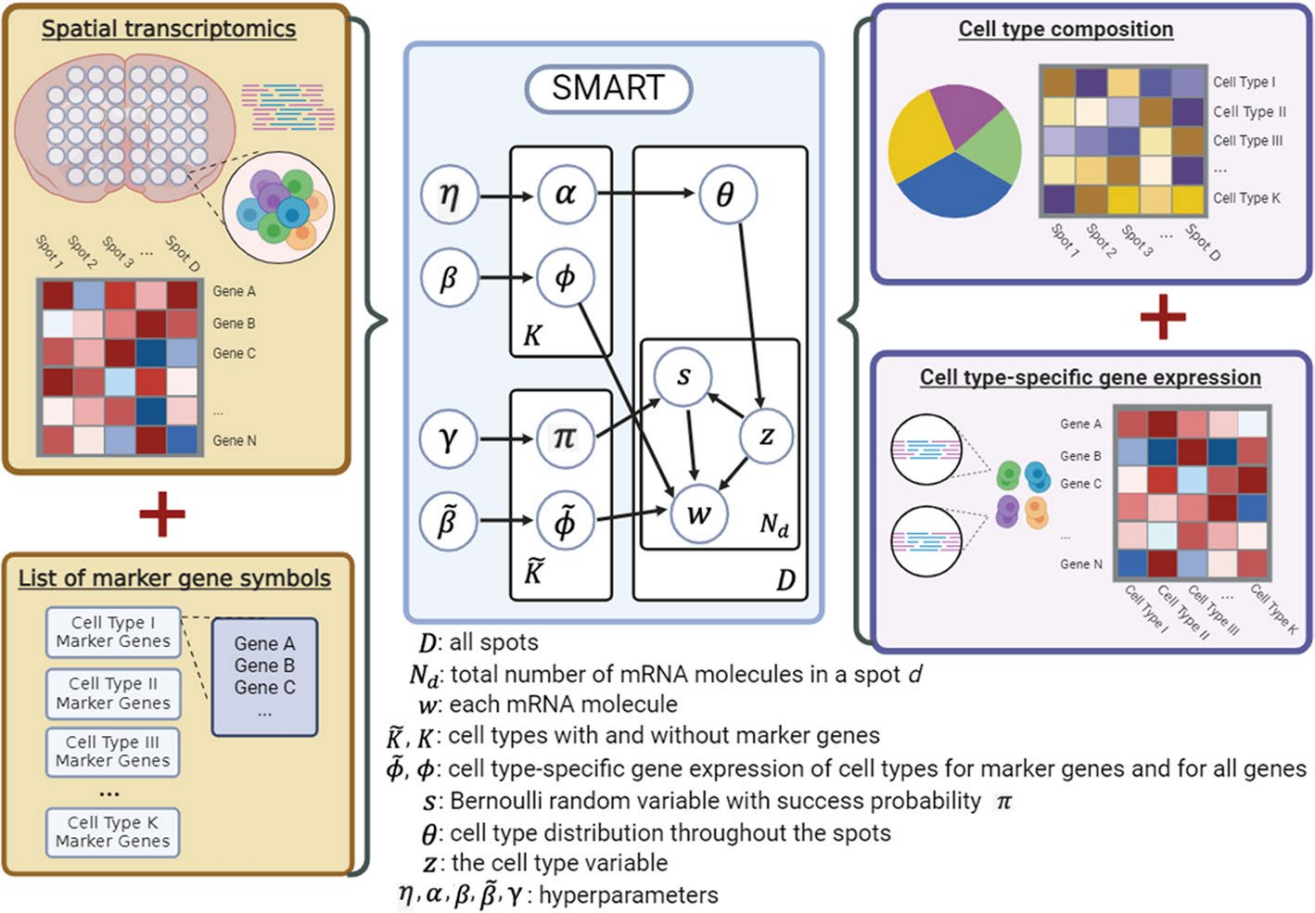
Overview of SMART. SMART takes the spatial transcriptomics data (a gene-by-spot matrix) and a list of marker gene symbols for each cell type as the inputs. Then, SMART uses a semi-supervised topic model to predict the cell type composition (a cell type-by-spot matrix) and the cell type-specific gene expression (a gene-by-cell type matrix) simultaneously. Both the cell type proportions $\theta$ and the cell type-specific gene expression are modeled as Dirichlet distributions. Assuming marker genes have a higher expression than non-marker genes in a cell type $k$, the final cell type-specific gene expression is modeled as a mixture of two Dirichlet distribution ${\widetilde{\phi }}_{k}$ for marker genes and ${\phi }_{k}$ for all genes, so that the marker genes have higher prior means than the non-marker genes. Controlling by the Bernoulli variable ${s}_{di}$ for the $i$ th molecule in spot $d$, the mRNA molecule ${w}_{di}$ is sampled based on either ${\widetilde{\phi }}_{k}$ or ${\phi }_{k}$. The model is described in more detail in the “ Methods” section

To address these challenges, we present *S*patial transcriptomics deconvolution using *MAR*ker-gene-assisted *T*opic model (SMART), a marker-gene-assisted deconvolution method based on semi-supervised topic models (Fig. 1). In natural language processing, the topic models were used to identify the topic distribution from the words in a large number of unlabeled documents, as well as the word frequencies within each topic. In the context of ST deconvolution, SMART simultaneously infers the cell type composition of the spots and the cell type-specific gene expression profile. Compared to unsupervised approaches such as STdeconvolve, which uses the marker information after the deconvolution process to label the latent topics, SMART directly incorporates marker gene information as prior knowledge during the topic inference procedures to guide cell type identification and, thus, improves the predictive accuracy and minimizes the variability. Using three datasets simulated from single-cell ST data and two real ST datasets, we demonstrate that SMART accurately estimates cell type composition and cell type-specific gene expression. Without needing a reference dataset, it outperforms some of the best-performing reference-based and reference-free methods for ST data [16–18] when an ideal reference dataset is unavailable. Instead of a scRNA-seq reference, SMART uses a list of marker gene symbols for each cell type as the input. SMART also allows the inclusion of cell types with no marker gene information (“no-marker” cell types), which can be helpful in identifying novel cell types. The performance of SMART on cell subtypes can be augmented using a two-stage approach. SMART also provides condition-specific estimates with a covariate model, elucidating molecular changes across different biological conditions.

## Results

### SMART accurately predicted cell type composition and cell type-specific gene expression in simulated ST data

**Fig. 2:**
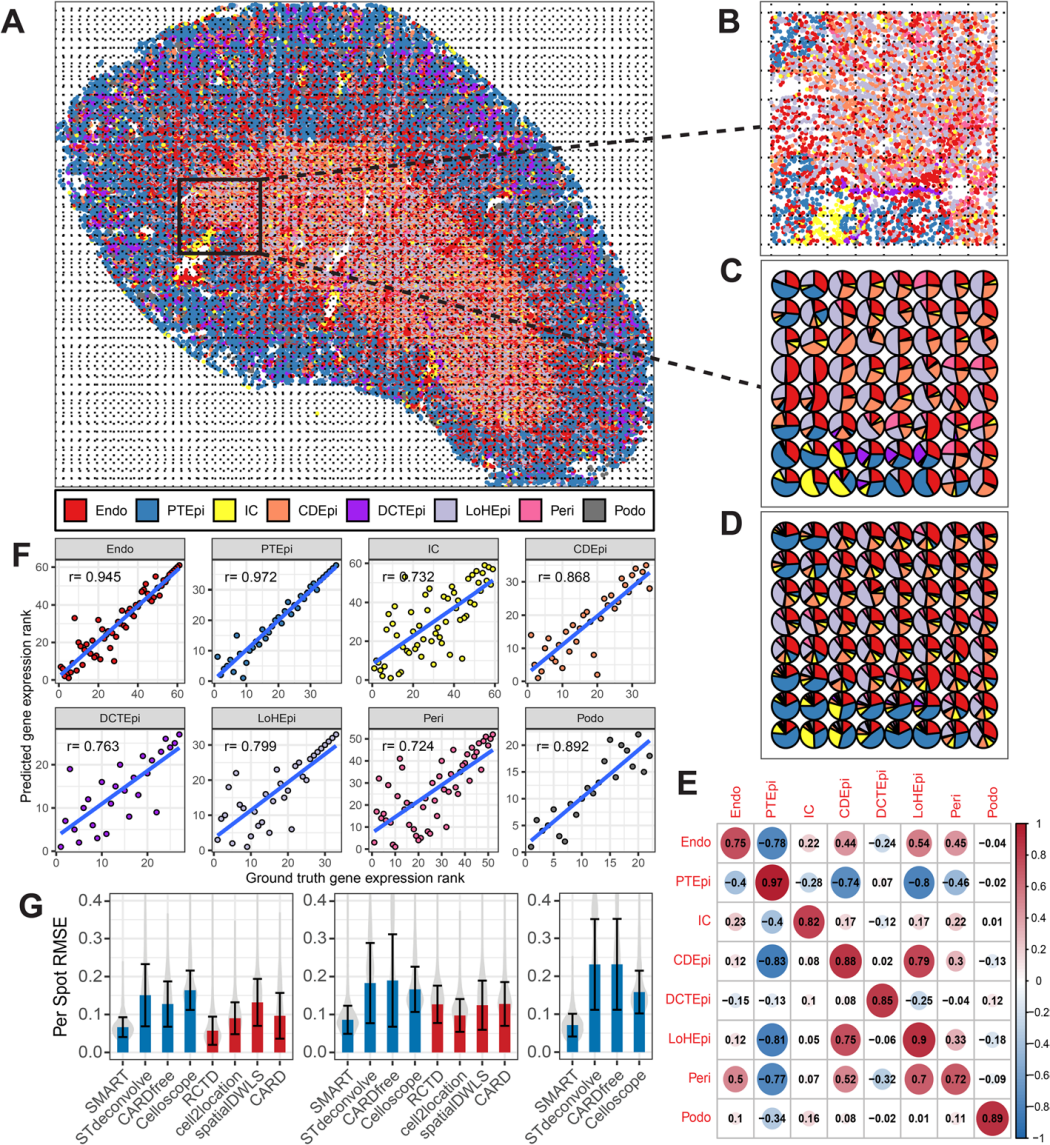
Performance evaluation of SMART using simulated ST data in mouse kidney. **A** Spatial image of the single-cell MERFISH data. **B** A zoom-in view of a selected area. **C** The cell type composition of the selected area. **D** The SMART-predicted cell type composition of the selected area. **E** A heatmap showing the Pearson correlation coefficients between the predicted and the GT cell type proportions of each cell type. **F** The Pearson correlation between the gene rank of the ground truth gene expression and the gene rank of the predicted gene expression for each cell type. Genes with relative gene count frequency < 0.1% were excluded due to low expression. **G** The per-spot RMSE between the predicted and the GT cell type proportions using the GT markers/reference (left), the TMS markers/reference (middle), and literature-based markers (right). Blue = marker-gene-assisted/reference-free methods; Red = reference-based methods. Abbreviations: endothelial cell (Endo), epithelial cell of the proximal tubule (PTEpi), immune cell (IC), collecting duct epithelial cell(CDEpi), distal convoluted tubule epithelial cell (DCTEpi), loop of Henle epithelial cell (LoHEpi), pericyte (Peri), podocyte (Podo)

To evaluate the performance of SMART, we used publicly available single-cell ST data in mouse kidneys (MK) [19], which were profiled using the Vizgen Multiplexed Error-Robust Fluorescence in situ Hybridization [20] (MERFISH) platform. The MK dataset contains the expression of 304 genes from 126,241 cells annotated to eight cell types. We simulated ST data by dividing the single-cell ST data of the MK dataset into 2474 spatially contiguous squares and aggregated the gene expression of cells within each square to mimic the spots of ST data (Fig. 2A and B). The ground truth (GT) cell type proportion, cell type-specific gene expression, and marker genes can be obtained accordingly from the simulated ST data and the original single-cell ST data (Fig. 2C). Then, we applied SMART to the simulated data along with the GT marker genes to simultaneously infer the cell type composition at each spot (Fig. 2D) and the cell type-specific gene expression profile. We observed a strong correlation (> 0.70) between the predicted cell type composition and the ground truth cell type composition in all cell types (Fig. 2E). Additional file 1: Fig. S1 shows the top 10 genes based on the predicted relative gene frequencies for each cell type. The top 10 genes in each cell type were significantly enriched with marker genes used for deconvolution (Fisher’s exact test *P* < 0.001). We also observed a strong correlation (> 0.70) between the rank of the genes in the GT gene expression profile and that in the predicted gene expression profile for each cell type (Fig. 2F).

### SMART demonstrated superior performance to existing methods in realistic settings

To compare the performance of SMART against existing deconvolution methods, we next applied some of the best-performing reference-free/marker-gene-assisted (STdeconvolve, CARDfree, Celloscope) and reference-based methods (RCTD, CARD, cell2location, spatialDWLS) [16–18] to the simulated MK dataset. Since STdeconvolve still needs to use marker genes to label the inferred cell types, in this benchmarking analysis, we considered it a marker-gene-assisted method. We first assessed the methods in an ideal situation where a reference dataset with the same biological and experimental detail is available. For reference-based methods, the original single-cell ST dataset used to simulate the ST data was used as the reference dataset; for marker-gene-assisted methods, the GT marker genes derived from the original single-cell ST dataset were used to infer the cell types to make the results more comparable to reference-based methods. We quantified the performance of each method with the Pearson correlation coefficient (PCC) and the root mean square error (RMSE) between the predicted and the GT cell type proportions of all spots across all cell types, as well as the per-spot RMSE between the predicted and the GT cell type proportions at each spot. In the simulated MK dataset, SMART demonstrated better performance than most of the methods (mean per-spot RMSE = 0.0666, PCC = 0.937, RMSE = 0.0715, Diebold-Mariano *P* < 0.001) except for RCTD (mean per-spot RMSE = 0.0572, PCC = 0.955, RMSE = 0.0565) (Fig. 2G left, Additional file 1: Fig. S2A left, and Additional file 1: Fig. S2B left), which showed slightly better performance than SMART. However, using the original single-cell ST dataset as the reference profile to predict the ST dataset simulated from it will likely provide the best possible results for reference-based methods. Therefore, the reference-based methods were given a testing advantage over the marker-gene-assisted methods. In reality, such an ideal reference profile with the same cell types and technical details rarely exists.

To examine the performance of the above methods in a more realistic situation where an ideal reference dataset is unavailable, we collected single-cell RNA-seq data in mouse kidneys from the Tabula Muris Senis (TMS) cell atlas [21]. The TMS single-cell dataset was used as the reference profile for reference-based methods to deconvolve the simulated MK dataset; the marker genes derived from the TMS dataset were used for marker-gene-assisted methods to make the results comparable to reference-based methods. In this situation, SMART demonstrated the best performance over all the other methods (per-spot RMSE = 0.0860, PCC = 0.921, RMSE = 0.0786, Diebold-Mariano *P* < 0.001, Fig. 2G middle, Additional file 1: Fig. S2A middle, and Additional file 1: Fig. S2B middle).

Finally, to demonstrate the advantage of SMART as a marker-gene-assisted method that does not rely on any reference datasets, we collected marker gene symbols from the existing literature [22] and the CellMarker 2.0 database [23]. Next, we apply the marker-gene-assisted methods with these marker genes. We showed that SMART demonstrated the best performance among the four marker-gene-assisted approaches (mean per-spot RMSE = 0.0712, PCC = 0.924, RMSE = 0.0772, Diebold-Mariano *P* < 0.001, Fig. 2G right, Additional file 1: Fig. S2A right, and Additional file 1: Fig. S2B right).

In all three scenarios, SMART demonstrated the smallest variability in per-spot RMSE (Fig. 2G). Additional file 1: Fig. S3 and Additional file 1: Fig. S4 compare the PCC and RMSE in each cell type in all three scenarios. The three sets of marker genes that we used for SMART deconvolution are shown in Additional file 2: Table S1, and Additional file 1: Fig. S5 shows the overlap among these three sets of marker genes. To ensure that SMART works with high-resolution platforms such as 10X Visium, we re-simulated the dataset to contain, on average, 10 cells per spot and repeated the benchmarking as a sensitivity analysis. We obtained similar results, and SMART showed the best performance when an ideal reference dataset was not available (Additional file 1: Fig. S6).

### SMART demonstrated improved stability and interpretability than unsupervised methods

Similar to other marker-gene-assisted methods based on generative models with sampling algorithms (i.e., STdeconvolve, Celloscope), the results of SMART may vary between runs according to the starting value. STdeconvolve, as an unsupervised approach, only uses marker genes after the deconvolution process to label the latent cell types, and sometimes, the results between runs can be completely different. In most cases, it can also be difficult to get an estimate for every cell type, particularly for rare cell types.

Both SMART and Celloscope are semi-supervised methods that use marker genes as prior knowledge during the deconvolution process to help improve prediction accuracy, stabilize performance, and make results more reproducible. To assess the variability in performance, we performed 100 repeats of SMART, Celloscope, and STdeconvolve on the MK dataset and examined the PCC and the RMSE between the predicted and GT cellular composition across all spots. As expected, we observed that both SMART and Celloscope delivered more consistent results than STdeconvolve with less variability (Additional file 1: Fig. S7A and S7B). To further improve the stability, SMART provides the option to perform a user-specified number of repeats in parallel and average the results. Additional file 1: Fig. S8 shows the PCC and the RMSE overall repeated runs in each cell type. Importantly, in many cases, STdeconvolve identified multiple latent topics for abundant cell types while identifying no topics for cell types that are less abundant. Out of the 100 repeats of STdeconvolve, 12 repeats identified only three cell types when using the GT marker genes in the GSEA to label the cell types; 58 repeats failed to identify more than five cell types; only four repeats were able to identify six cell types and no repeats identified all eight cell types. This indicates that the use of marker genes in SMART not only stabilizes the deconvolution process but also ensures that we obtain an accurate estimate for each cell type.

### SMART demonstrated improved performance with a two-stage approach

Although marker genes can be shared across cell types in SMART, we recommend using marker genes with high specificity to achieve the best results. However, in some cases, marker genes can be very similar between cell types (i.e., monocyte, macrophage, and dendritic cells), especially those arising from the same lineage [24], which makes it difficult to select specific marker genes. This ambiguity may lead to a drop in the accuracy of deconvolution results. To mitigate this limitation, we implemented a two-stage approach to improve the performance in predicting individual cell subtypes. In this two-stage approach, SMART was first applied to deconvolve the ST dataset into major cell types. Next, we extracted the gene counts explained by the cell type of interest. A second round of deconvolution was performed on the extracted gene counts to further decompose the cell type of interest to its subtypes. By separating the deconvolution process into two stages, any non-specific marker genes shared between the major cell types and those that were discarded in the first stage may be re-used in the second stage to discriminate the cell subtypes. In this manner, the selection of marker genes becomes easier for subtype identification compared to a one-stage approach that estimates all major cell types and cell subtypes at once.

**Fig. 3:**
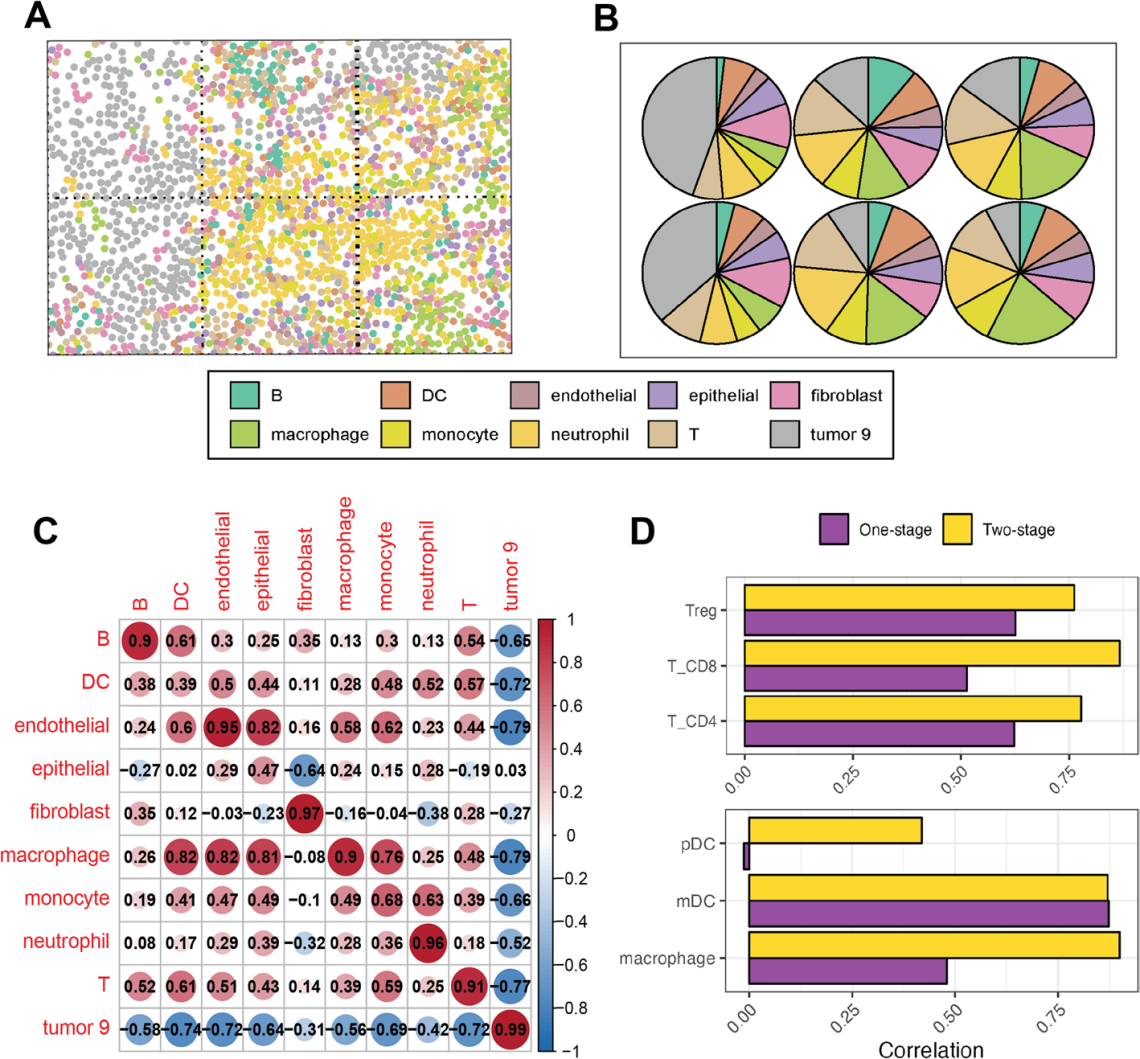
Demonstration of the two-stage approach of SMART. **A** An example field of view of the single-cell Nanostring CosMx data in human non-small cell lung cancer. **B** The SMART-predicted cell type composition of the example field of view. **C** A heatmap showing the Pearson correlation coefficients between the predicted and the GT cell type proportions of each cell type. **D** A comparison between the one-stage approach and the two-stage approach on predicting T cell subtypes (top) and dendritic cell subtypes (bottom)

To illustrate, we collected a human non-small cell lung cancer (NSCLC) single-cell ST dataset [25], which was profiled using the NanoString CosMx platform and simulated a ST dataset of 120 contiguous spots in the same manner (Fig. 3A). The NSCLC single-cell dataset contains the expression of 960 genes from 32,272 cells. These cells were pre-annotated to ten major cell types or thirteen cell types with the inclusion of T cell subtypes (CD4 + T, CD8 + T, regulatory T) and dendritic cell (DC) subtypes (plasmacytoid DC and myeloid DC). In the two-stage approach, we first applied SMART to deconvolve the ST dataset into the major cell types (Fig. 3B). Most cell types demonstrated a PCC > 0.90 between the GT and the predicted cell type composition (Fig. 3C). Next, we extracted the gene counts explained by the T cells to further deconvolve them into T cell subtypes. The T cell subtype proportions obtained from the two-stage approach showed stronger correlations with the GT T cell subtype proportions compared to a regular one-stage approach (Fig. 3D top). Users can also choose to include a cell type that is transcriptomically similar to the cell type of interest in the second round of deconvolution to recover potential incorrectly allocated gene counts. For example, to assist in the identification of DC subtypes, we included macrophages, which showed the greatest similarity to DCs in gene expression, during the second-stage deconvolution of DCs. By doing this, the correlation between the GT and predicted cell type proportion showed a dramatic increase for both pDCs and macrophages while staying similar for mDCs (Fig. 3D bottom). The marker genes used for deconvolution of the major cell types, T cell subtypes, and DC subtypes are shown in Additional file 3: Table S2.

### SMART identified condition-specific genes for each cell type with a covariate model

**Fig. 4:**
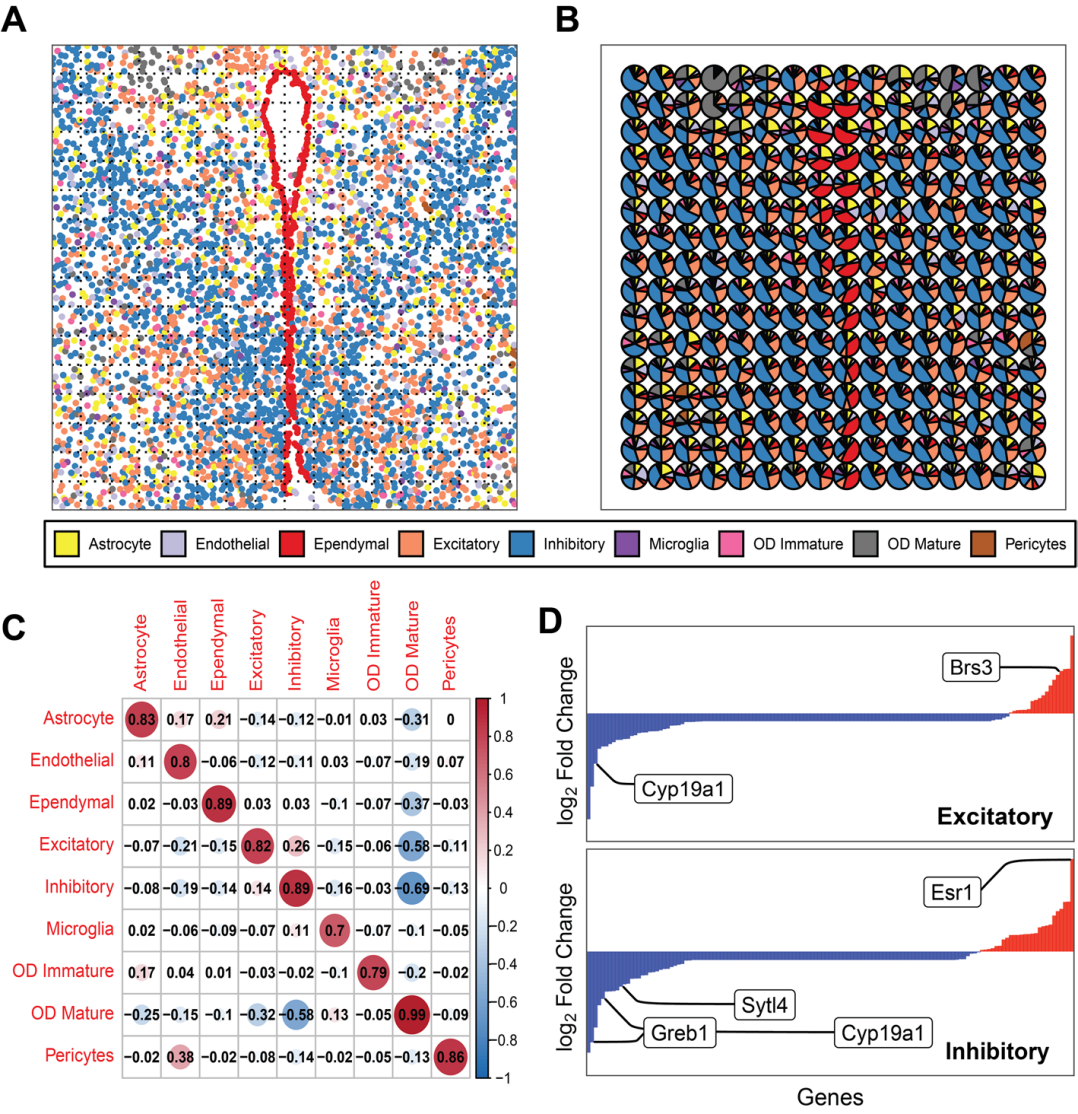
Demonstration of the covariate model of SMART using simulated data in mouse hypothalamic preoptic region (*N* = 6 with 3 female mice and 3 male mice). **A** Spatial image of an example slice of the single-cell MERFISH data. **B** The SMART-predicted cell type composition for the example slice. **C** A heatmap showing the Pearson correlation coefficients between the predicted and the GT cell type proportions of each cell type. **D** The log_2_ fold change of gene expression between the female mouse and the male mouse in the excitatory neurons (top) and the inhibitory neurons (bottom). Red bars = genes up-regulated in the female mice; Blue bars = genes up-regulated in the male mice

While reference-based methods assume that the cell type-gene expression is constant regardless of the sample conditions and that only the cell type composition can be changed, SMART respects the fact that cell type-specific gene expression can also vary between conditions. This was achieved by allowing users to incorporate covariates to identify condition-specific gene signatures for each deconvolved cell type. For demonstration, we simulated a ST dataset using a single-cell ST dataset on mouse hypothalamic preoptic area (MPOA) generated using the MERFISH platform [26] (Fig. 4A). The MPOA dataset contains the expression of 135 genes from 188,658 cells of 6 mice (3 female and 3 male). We applied the covariate model using sex as the covariate and obtained the sex-specific gene expression for each cell type and the cell type proportions (Fig. 4B). Again, to avoid any confounding factors that may affect the evaluation of the model, the GT marker genes were used with the covariate model of SMART (Additional file 4: Table S3). We observed a strong correlation of > 0.7 between the GT and the predicted cell type proportions in every cell type (Fig. 4C). Consistent with the literature, we observed that in excitatory neurons, Brs3 was up-regulated in the female mouse, and Cyp19a1 was up-regulated in male mice [26] (Fig. 4D top); in inhibitory neurons, Esr1 was up-regulated in female mice while Sytl4, Cyp19a1, and Greb1 were up-regulated in male mice [26] (Fig. 4D bottom). We then incorporated the effect sizes of the sex-specific gene expression profile with GSEA, identifying pathways that were enriched due to sex differences within each cell type. For example, we found that the late estrogen response was up-regulated in the excitatory neurons of female mice, and the transmembrane transporter activity was up-regulated in the microglia of female mice in comparison with those of the male mice (Benjamini–Hochberg false discovery rate < 0.05).

### SMART is compatible with diverse ST platforms

**Fig. 5:**
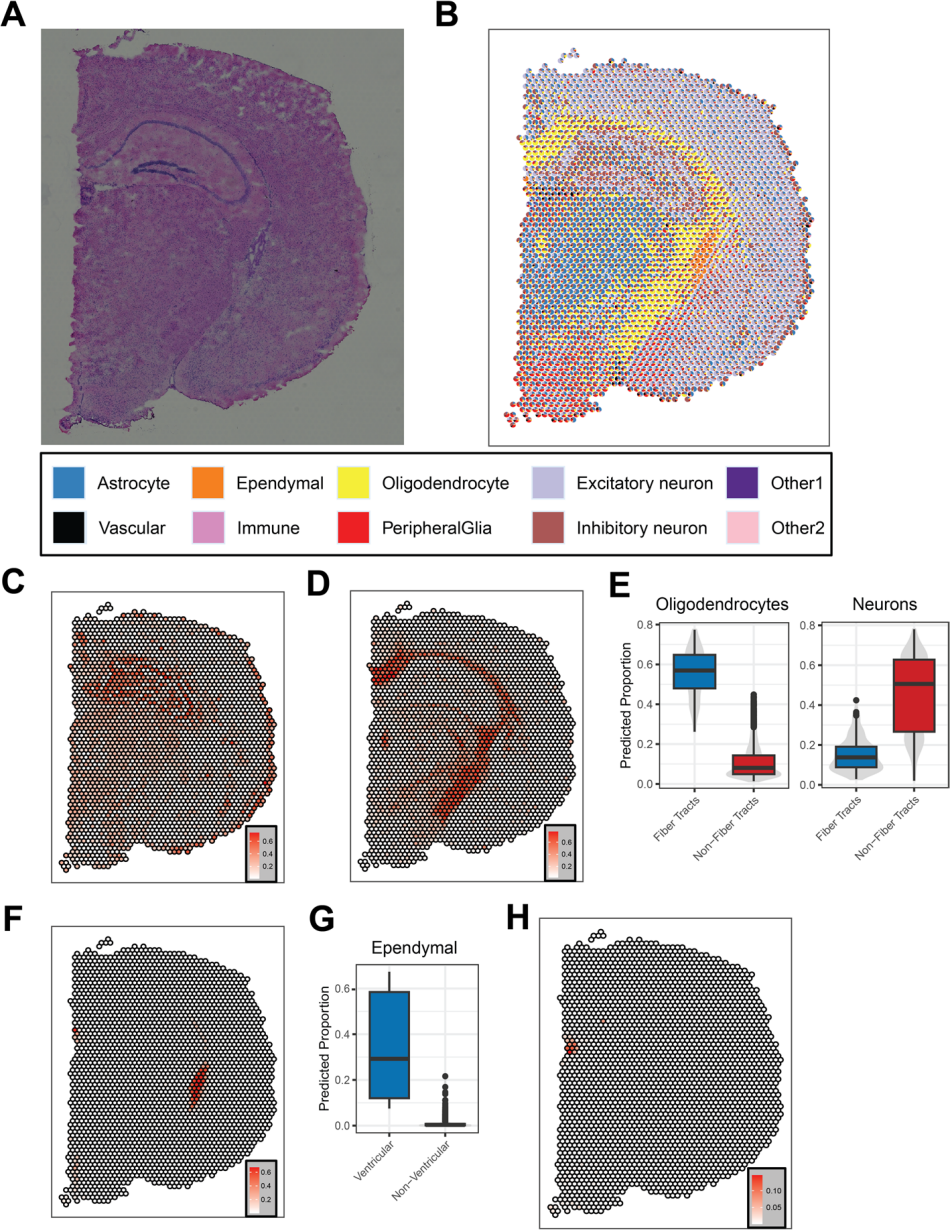
Application of SMART on a mouse brain ST dataset profiled using the 10X Visium platform. **A** Histology staining image of the tissue. **B** SMART-predicted cell type composition. **C** The predicted proportion of inhibitory neurons in each spot. **D** The predicted proportion of oligodendrocytes in each spot. **E** A comparison of the predicted proportion of oligodendrocytes (left) and neurons (right) between the spots in the fiber tract region and the spots in the non-fiber tract region. **F** The predicted proportion of ependymal cells in each spot. **G** A comparison of the predicted proportion of ependymal cells between the spots in the ventricular region and the spots in the non-ventricular region. **H** The predicted proportion of an unknown cell type in each spot

To validate the performance of SMART on real ST datasets, we applied SMART to a mouse brain ST dataset [27], which was profiled using the 10X Visium platform (Fig. 5A). Depending on the tissue type, the Visium platform usually contains 1–10 cells per spot [3]. With marker genes identified from the Mouse Brain Atlas [28] and the CellMarker 2.0 database, we deconvolved the mouse brain ST dataset into seven major cell types. Then, we used the two-stage model to further deconvolve the neurons into excitatory and inhibitory neurons (Fig. 5B). In both stages, we included an unknown cell type to represent any novel cell types or cell types that cannot be explained by the specified cell markers. SMART successfully identified cell types in brain regions such as fiber tracts, ventricles, cortex, thalamus, and hypothalamus. The regions were annotated based on the anatomical images from the Allen Brain Atlas [29, 30]. The distribution of the excitatory neurons and the inhibitory neurons in the cortex was consistent with literature that the cortical layer 1 (the outer layer) contains mainly the inhibitory neurons and that the excitatory neuron is overall more abundant than the inhibitory neurons (Fig. 5C) [31, 32]. Also, as expected, the oligodendrocytes were predicted to be highly enriched in fiber tracts compared to non-fiber tract regions [33] (*t*-test *P* < 0.001, Fig. 5D and E); neurons were highly enriched in non-fiber tract regions as opposed to fiber tracts (*t*-test *P* < 0.001, Fig. 5E). Similarly, we observed a high predicted proportion of ependymal cells, which form an epithelial lining for the brain ventricles [34], in ventricular regions compared to non-ventricular regions (*t*-test *P* < 0.001, Fig. 5F and G). Interestingly, the unknown cell type we obtained during the first stage may correspond to a cell type in the medial habenula region of the mouse brain (Fig. 5H). This suggests that SMART may help identify novel cell types when marker genes are not provided.

**Fig. 6:**
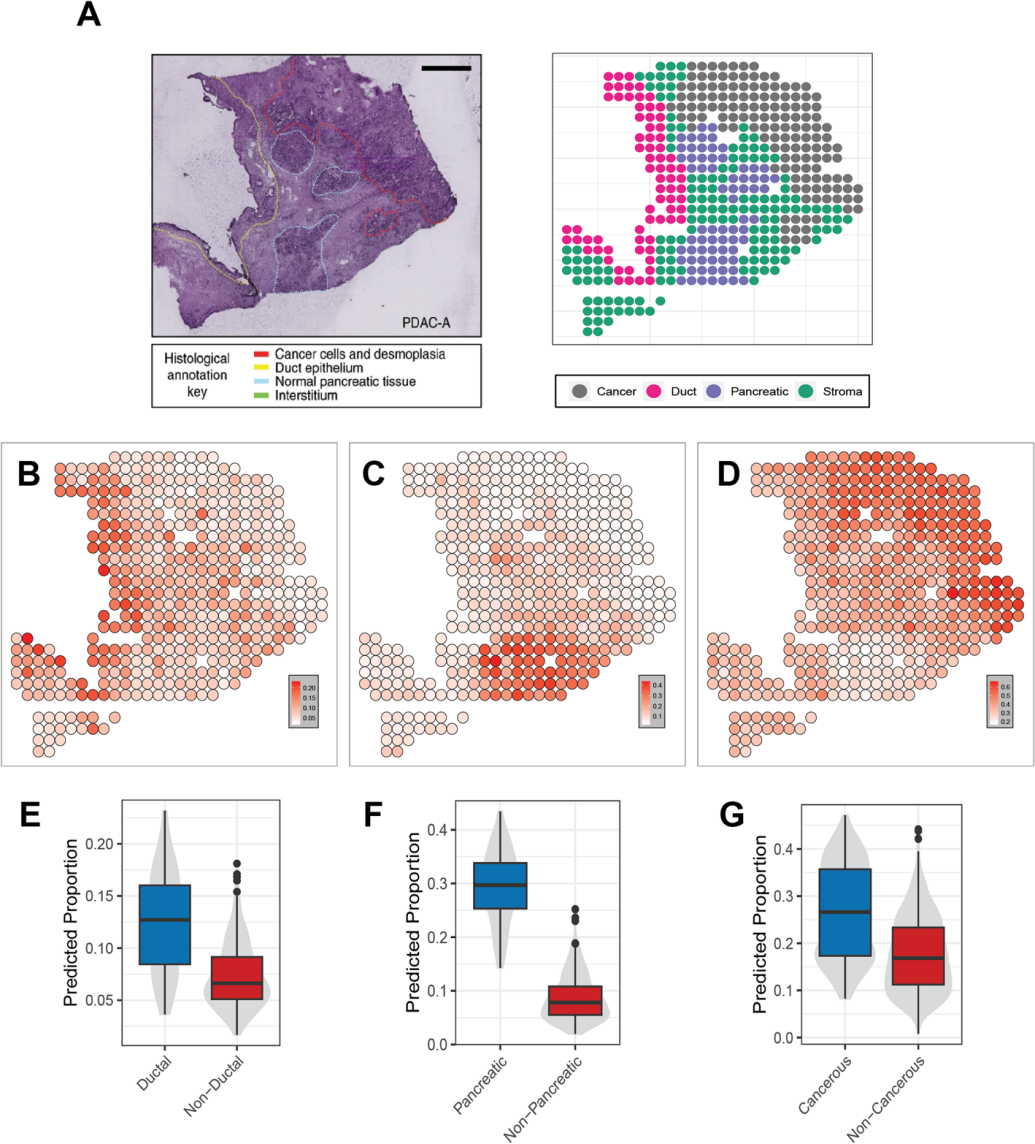
Application of SMART on a pancreatic ductal adenocarcinoma ST dataset. **A** Histology staining image of the tissue (left) and the regions annotated by histologists from the original study (right). **B** The predicted proportions of ductal cells. **C** The predicted proportions of acinar cells. **D** The predicted proportions of cancer clone cells. **E** A comparison of the predicted proportion of ductal cells in the spots of ductal region and the spots of the non-ductal region. **F** A comparison of the predicted proportion of acinar cells in the spots of the pancreatic region and the spots of the non-pancreatic region. **G** A comparison of the predicted proportion of cancer clone cells in the spots of the cancerous region and the spots of the non-cancerous region

In addition to the mouse brain ST dataset, we also validated the performance of SMART on an ST dataset generated from the human pancreatic ductal adenocarcinoma (PDAC) sample [35] using microarray slides (Fig. 6A). With marker genes derived from its sample-matched scRNA-seq dataset generated using the inDrop platform, SMART identified sixteen cell types across four distinct tissue regions labeled based on histology staining (Fig. 6B–D). As expected, we observed a higher predicted proportion of ductal cells in the ductal region than in the non-ductal regions (*t*-test *P* < 0.05, Fig. 6E). Also, we observed a higher predicted proportion of acinar cells in the pancreatic region [36] (*t*-test *P* < 0.05, Fig. 6F) and cancer clone cells in the cancerous region (*t*-test *P* < 0.05, Fig. 6G).

### Factors affecting the performance of SMART

Finally, to assess factors that may affect the results of SMART, we first examined whether the total number of spots affects the deconvolution performance. With the simulated MK dataset, we randomly selected 50, 100, 300, 500, and 1000 spots and applied SMART. The results show that interrogation of more spots did not lead to a significant decrease in the per-spot RMSE (*t*-test *P* > 0.05, Additional file 1: Fig. S9A).

Next, we examined if the number of cells in each spot has any effect on SMART. With technological improvement, the resolution has become higher in recent ST platforms [37]. Using the NSCLC dataset, we re-simulated the ST data by decreasing the size of the contiguous squares to contain, on average, 269, 68, and 17 cells per spot to mimic the scenario that higher-resolution platforms usually have more spots with a smaller tissue coverage area in each spot. Interestingly, while the change in per-spot RMSE is subtle, it decreased with a higher number of cells per spot (*t*-test *P* < 0.001, Additional file 1: Fig. S9B), suggesting that SMART may perform better on ST platforms with a lower resolution.

Most importantly, since the selection of input marker genes can affect the performance of SMART, we checked the presence of marker genes in the top 10 SMART-predicted cell type-specific genes in the MK dataset when the GT, the TMS, and the literature-based marker genes were used as input (Additional file 5: Table S4). As expected, most of the top 10 predicted genes for each set were also the input marker genes. These marker genes contained both genes shared between the marker gene sets and unique marker genes in each set. Thus, these data indicate that different marker genes can be used with SMART, although the shared marker genes, which are considered more reliable, probably impose a greater impact on the overall results. Notably, the top 10 genes also contained non-marker genes, indicating the robustness of SMART to the inclusion of marker genes that were of reduced quality or missing altogether from the input, so long as the overall proportion of such genes is relatively small. Next, we examined how the number of marker genes affects the results of SMART by using at most 3, 5, 10, and 15 marker genes per cell type. As anticipated, SMART demonstrated a lower per-spot RMSE as the number of marker genes increased (*t*-test *P* < 0.001, Additional file 1: Fig. S9C). This suggests that including more marker genes improves the performance of SMART, assuming the quality of the marker genes. The largest decrease in mean per-spot RMSE occurred between having five marker genes and having ten marker genes (7.13% decrease). These data suggest that having approximately ten marker genes per cell type may efficiently improve the performance of SMART.

The runtime of SMART increases proportionally with the total library size and the number of deconvolved cell types. Thus, for genome-wide datasets, we recommend including a gene selection procedure to keep only the marker genes and highly variable genes to reduce the runtime. In Additional file 1: Fig. S10, we benchmarked the runtime of SMART on randomly selected spots from the MK simulated dataset.

### Compare SMART with bulk transcriptomics deconvolution methods

Although SMART does not use spatial information for deconvolution, it works best on ST data. To compare the performance of SMART against some of the best-performing bulk transcriptomics deconvolution methods [38, 39], in addition to the main benchmarking analysis (Fig. 2), we also applied Cibersort [40], MuSiC [41], BayesPrism [42], DWLS [43], SCDC [44], Bisque [45], and GTM-decon [46] to the simulated MK ST dataset. All seven methods were run with their default parameters. Since all seven methods are reference-based methods, the batch effect between the reference dataset and the ST dataset should be considered. Therefore, we performed the evaluation in a more realistic setting using the TMS dataset as the reference for Cibersort, MuSiC, BayesPrism, DWLS, SCDC, Bisque, and GTM-decon; marker genes derived from the TMS dataset were used with SMART. As expected, SMART outperformed all seven methods with a smaller per-spot RMSE (Diebold-Mariano *P* < 0.001), a smaller RMSE across all spots, and a stronger correlation across all cell types between the predicted and the GT cell type proportions (Additional file 1: Fig. S11), indicating that SMART is more suitable for ST datasets than the bulk transcriptomics deconvolution methods.

## Discussion

Using multiple simulated and real ST datasets, here, we demonstrated that SMART accurately captures the cell type composition and cell type-specific gene expression of ST data across various ST platforms, even in comparison with some of the best-performing reference-based methods [16–18]. Using the MK simulated dataset, we showed that in an ideal situation, where we used the original single-cell dataset as the reference to deconvolve the ST dataset simulated from it, SMART showed comparable and potentially better performance to the reference-based methods. Although methods such as RCTD may show a slightly better performance, such ideal conditions rarely exist. In a more realistic circumstance where we used an external reference dataset, SMART shows significantly lower error compared to reference-based methods, indicating that SMART, as a marker-gene-assisted tool, provides more accurate results in real-world settings. Without the need to properly process a reference dataset, SMART minimizes the impact of batch effects and makes it easier to use compared to most reference-based methods. We also demonstrated that, with marker genes collected from the literature and public marker gene databases, SMART achieved the best performance compared to other marker-gene-assisted methods. The performance was even comparable to the reference-based methods with an ideal reference. Compared to unsupervised topic model-based methods such as STdeconvolve, which only uses marker gene information after deconvolution to label the cell types, the results of SMART are also more reproducible by including prior knowledge on marker genes during the deconvolution process. This is also one of our motivations for developing SMART. The results from the NSCLC dataset showed that the two-stage approach might help to further improve SMART’s performance in decomposing cell subtypes by optimizing the use of marker genes and by recovering falsely allocated gene counts. Finally, we used the MPOA dataset to demonstrate the ability of SMART-covariate to estimate the condition-specific gene expression profile for each cell type by including the condition as a covariate. An important assumption in the reference-based methods is that the cell type-specific gene expression is constant across sample conditions, and only cell type compositions differ. However, this assumption is frequently violated in the real world as gene expression in samples is modified by disease and treatment, leading to inaccurate deconvolution results. SMART surmounts this limitation by enabling the inclusion of covariates that can capture the impact of disease status and phenotypes. The covariate model can also be combined with the two-stage approach to obtain condition-specific estimates for the cell subtypes, and where appropriate, different sets of covariates may be used at each stage to estimate the cell subtypes more precisely. In addition, the ability to include cell types without marker genes assists in identifying novel cell types, as shown in the analysis of the 10X Visium mouse brain dataset. With the two real ST datasets, we showed the compatibility of SMART on high-resolution platforms such as the 10X Visium as well as lower-resolution platforms such as the microarray platform used in the PDAC dataset.

SMART allows shared marker genes between cell types; however, using markers specific to cell types usually provides better results. Although the deconvolution results may not be significantly affected even if a small portion of marker genes are of reduced quality or are missing from the input, we recommend that the users consult multiple sources to identify and select the most reliable marker genes (i.e., marker genes that have been confirmed by multiple sources). While obtaining high-quality marker genes can be challenging, SMART only requires a small number of marker genes. As illustrated with the NSCLC dataset, we can obtain a good to excellent estimation with as few as three marker genes per cell type. The results can be improved efficiently by using approximately ten marker genes per cell type.

Lastly, we demonstrated that SMART is more suitable for ST data compared to bulk transcriptomics deconvolution methods. Similar to other topic model-based methods for ST data, SMART assumes that the proportional distribution of cell types across spots is heterogeneous and sparse (i.e., some spots have more of cell-type A and others have more of cell-type B). Spatial transcriptomics data, which only contains tens or hundreds of cells per spot, generally satisfies this assumption. In bulk transcriptomics data, however, the cell type composition is more homogeneous across the samples with much lower sparsity. For example, the 10X Visium platform contains only 1–10 cells on average, while bulk RNA-seq samples may contain hundreds of thousands of cells. Moreover, as a marker-gene-assisted method without needing a reference dataset, SMART works best when there are a sufficiently large number of spots (samples). This condition is also generally satisfied in ST data compared to bulk transcriptomics data. On the other hand, the bulk transcriptomics deconvolution methods are more specialized for bulk transcriptomics data and therefore, may not provide accurate estimates for ST datasets.

There are also limitations to SMART. Firstly, all the spots are assumed to be independent in SMART instead of borrowing spatial information from the adjacent spots. However, SMART does include a gene count weighting scheme to borrow gene abundance information from all spots to prevent certain genes from dominating a cell type. Moreover, the selection of input marker genes is critical to optimize the performance of SMART. To make the tool more accessible, SMART provides several pre-defined lists of marker genes for common tissue types.

## Conclusion

In summary, we present SMART as a marker-gene-assisted deconvolution method for spatial transcriptomics without needing a scRNA-seq reference profile. By incorporating marker genes for the cell types as guidance, SMART showed improved accuracy, stability, and interpretability even when compared with some of the best-performing reference-based methods. With the two-stage approach, SMART shows an advantage in discriminating the cell subtypes. The covariate model provides insight into the condition-specific gene expression of each cell type and may be helpful for studying biological perturbations. Ultimately, we believe that SMART will be a powerful tool to unravel the tissue heterogeneity and identify potential therapeutic targets at a single-cell-type resolution with spatial information.

## Methods

### Overview of SMART

SMART builds on the keyword-assisted topic models (keyATM) [47], which are semi-supervised topic models that integrate prior knowledge to guide the formation of topics. By including a small number of keywords for each topic prior to model fitting, keyATM accurately infers the proportion of topics within each document and the word frequencies within each topic.

In the context of cell type deconvolution in SMART, the spots correspond to the documents, the genes correspond to the words, and the cell types correspond to the inferred topics. A small number of marker genes for each cell type (keywords) were used as prior knowledge to help infer the cell type proportions (topic proportions within each document) and the cell type-specific gene expression (word frequencies within each topic) in the form of relative gene frequencies.

The ST data is represented as a $V\times D$ matrix with $V$ genes and $D$ spots. The total number of RNA molecules in each spot $d$ is ${N}_{d}$. We use ${w}_{di}$ to represent the $i$ th mRNA molecule in spot $d$. KeyATM is a generative model based on a mixture of two Dirichlet distributions, one for the marker genes only and one for all genes. A key assumption is that the marker genes should have a higher expression in a given cell type than the non-marker genes. The data generation process is as follows:

1) Suppose there are a total of $K$ cell types and the first $\widetilde{K}$ of them are cell types provided with marker genes.

2) For each mRNA molecule $i$ in spot $d$, we draw the cell type variable ${z}_{di}$ from the topic distribution:

$${z}_{di}\stackrel{indep.}{\sim }\text{Categorical }({\theta }_{d})$$

${\theta }_{d}$ represents the cell type proportions within each spot $d$.

3) If the sampled cell type $k$ is a “no-marker” cell type, we draw the mRNA molecule ${w}_{di}$ from the standard gene distribution:

$${w}_{di} | {z}_{di}=k \stackrel{indep.}{\sim }\text{Categorical }({\phi }_{k})$$

$${\text{for }} k\in \{ \widetilde{K}+1,\widetilde{ K}+2, \dots , K\}$$

${\phi }_{k}\stackrel{i.i.d.}{\sim }\text{Dirichlet}(\beta )$ represents the standard gene frequencies for all genes within cell type $k$ with $V$ dimensions.

4) If the sampled cell type contains marker genes, we first draw a Bernoulli random variable ${s}_{di}$ with success probability ${\pi }_{k}$ for mRNA molecule $i$ in spot $d$

$${s}_{di} | {z}_{di}=k \stackrel{indep.}{\sim }\text{Bernoulli }\left({\uppi }_{k}\right) {\text{for }} k\in \{ 1, 2, \dots , \widetilde{K}\}$$

$${\text{where }} {\pi }_{k}\stackrel{i.i.d.}{\sim }\text{Beta }\left({\upgamma }_{1},{\upgamma }_{2}\right) \, {\text{for }} k\in \{ 1, 2, \dots , \widetilde{K}\}$$

If the variable equals 1, the mRNA molecule ${w}_{di}$ is drawn from the gene frequencies for cell type $k$ with marker genes ${\widetilde{\phi }}_{k}\stackrel{i.i.d.}{\sim }\text{Dirichlet}(\widetilde{\beta })$; if the variable equals 0, ${w}_{di}$ is drawn from the standard gene frequencies for cell type $k$, ${\phi }_{k}$. That is,

$${w}_{di} | {{s}_{di},z}_{di}=k \stackrel{indep.}{\sim }\left\{\begin{array}{c}{\text{Categorical}} ({\phi }_{k}) \, {\text{ if }} \, {s}_{di}=0\\ {\text{Categorical}}\left({\widetilde{\phi }}_{k}\right) \, {\text{ if }} \, {s}_{di}=1\end{array}\right.$$

$${\text{for }} k\in \{ 1, 2, \dots , \widetilde{K}\}$$

${\widetilde{\phi }}_{k}$ is a $V$ dimensional vector with positive values for the marker genes and zeros for the non-marker genes. $\beta$ and $\widetilde{\beta }$ are hyperparameters that make the prior means for the frequency of marker genes higher than those of non-marker genes.

5) And,

$${\theta }_{d}\stackrel{i.i.d.}{\sim }\text{Dirichlet}\left(\alpha \right) \text{ for }d=\text{1,2},\dots ,D$$

$$\alpha \stackrel{indep.}{\sim }\left\{\begin{array}{c}{\text{Gamma}} \left({\widetilde{\eta }}_{1},{\widetilde{\eta }}_{2}\right) \, {\text{for }} k\in \{ 1, 2, \dots , \widetilde{K}\} \, \\ {\text{Gamma}} \left({\eta }_{1},{\eta }_{2}\right) \, {\text{for }} k\in \{ \widetilde{K}+1,\widetilde{ K}+2, \dots , K\}\end{array}\right.$$

${\widetilde{\eta }}_{1}$ and ${\widetilde{\eta }}_{2}$ were set to sample $\alpha$ from smaller values so that the spots are more dominant by cell types with marker genes.

Figure 1 shows a graphic representation of this generative process. By integrating out the latent variables $(\theta , \phi , \widetilde{\phi }, \pi )$, keyATM uses a collapsed Gibbs sampling algorithm to sample from their posterior distribution. It also uses an inverse gene frequency weighting strategy to help prevent highly expressed genes from dominating the inferred cell types. The resulting $\theta$ matrix represents the cell type proportions for each spot; a final gene frequency ${\phi }_{k}^{*}$ combining $\phi$ and $\widetilde{\phi }$,, represents the cell-type specific gene expression, and is given by

$${\phi }_{kv}^{*}=\left(1-{\pi }_{k}\right){\phi }_{kv}+{\pi }_{k}{\widetilde{\phi }}_{kv}$$

for each gene $v$. More information regarding the sampling algorithm is available in Additional file 1: Supplementary Methods.

### The two-stage approach

To better estimate the composition of cell subtypes of a major cell type $k$, we take SMART one step further to perform a two-stage deconvolution as follows:

1. Deconvolve the spatial transcriptomics data into major cell types with the base model of SMART.
2. Calculate the total library size (sum of the gene counts) at each spot ${N}_{d}$.
3. Calculate the library size for the cell type of interest $k$ at each spot ${N}_{dk} = {N}_{d}{\theta }_{dk}$, where ${\theta }_{dk}$ is the cell type proportion of cell type $k$ of a spot $d$.
4. Calculate the counts for each gene in cell type $k$ at each spot $d$ with ${N}_{dk}{\phi }_{k}$, where ${\phi }_{k}$ is the relative gene frequency for cell type $k$.
5. Identify another cell type $m$ with the highest similarity by calculating the PCC or the Euclidian distance between the relative gene frequency of the cell types. This cell type $m$ can also be user-defined.
6. Calculate the gene counts for cell type $m$ at each spot $d$ as in steps 3 and 4.
7. Perform the second round of deconvolution on the sum of the gene counts for cell type $k$ and $m$. By including the cell type $m$, we aim to recover any gene counts that were potentially misclassified in stage one.

The two-stage approach includes a “no-marker” cell type in both stages to represent any data that cannot be explained by the cell types with marker genes.

### The SMART-covariate model

SMART-covariate extends the base model and builds on the keyATM covariate model. Instead of step 5) in the base model, the covariate model uses the following cell type distribution:

$${\theta }_{d}\stackrel{indep.}{\sim }\text{Dirichlet}\left(\text{exp}({{\varvec{\uplambda}}}^{T}{\mathbf{x}}_{\text{d}}\right)) \text{ for }d=\text{1,2},\dots ,D$$

$$\text{where }{\lambda }_{mk}\stackrel{i.i.d.}{\sim }N(\mu , {\sigma }^{2})$$

${\text{x}}_{\text{d}}$ is an $M$-dimensional covariate matrix for each spot $d$. ${\varvec{\uplambda}}$ is an $M\times K$ matrix of coefficients and ${\lambda }_{mk}$ is the ($m,k$) element of ${\varvec{\uplambda}}$**.**

### Simulating ST data

To mimic the spots of ST data, we collected pre-annotated single-cell resolution ST data and cut the spatial image into contiguous squares. To start with, the bottom and left edges of the bottom-left square were aligned with the bottom and the left edge of the spatial image. Then, the squares were created by drawing boundaries in increments of a selected value based on the datasets until they reached the top and right edge of the spatial image. Any squares with less than two cells were removed from the simulated dataset. Squares overlapping with the spatial image's edges were also removed. The gene counts of cells within the coordinate of each square were aggregated together to obtain the gene expression of this simulated spot. The GT cell type proportions at each spot can be calculated by counting the number of cells of each cell type in each square. The GT cell type-specific gene expression can be obtained by averaging the gene expression of cells of the same cell type in the original single-cell ST dataset. Finally, the GT gene markers for each cell type can be obtained through a differential expression analysis between one cell type and the rest. The differential expression analysis was performed with a Wilcoxon rank-sum test, and the Benjamini–Hochberg procedure was used to correct for multiple hypotheses testing. A false discovery rate < 0.05 was used as the threshold for significant marker genes. The resulting markers were also filtered by fold changes > 2 to select markers of high confidence. The marker genes were further pruned to keep marker genes specific to each cell type. Marker genes from the CellMarker 2.0 database were used if no GT marker genes were identified at the set threshold.

### Deconvolution using SMART and existing methods

To make the comparison between marker-gene-assisted methods and reference-based methods more comparable, the single-cell ST data used to simulate the ST datasets were used as the reference profile for reference-based methods, and the marker genes identified from the reference datasets were used for marker-gene-assisted methods. For the MK dataset, an external TMS reference dataset was also used to evaluate the deconvolution performance when an ideal reference is unavailable.

In SMART, the input ST data could be either gene counts or un-transformed normalized gene expression rounded to integers. A list of gene symbols for marker genes of each cell type was used as the supplemental input to guide cell type inference. By including the marker genes prior to deconvolution, the resulting cell type proportions and cell type-specific gene expression were automatically labeled with cell type names, improving the results’ interpretability. In addition to cell types with marker genes, SMART also allows the inclusion of unknown cell types without specifying any marker genes, which may be helpful in identifying novel cell types. The GSEA in SMART-covariate was performed using the R package “fGSEA” [48].

As an unsupervised reference-free method, STdeconvolve used the ST data as the only input. The resulting cell type proportions and cell type-specific gene expression matrices contained no cell type names. These unlabeled cell types were subsequently annotated with a name through a GSEA by comparing the inferred cell type-specific gene expression profile against the marker genes using the R package “liger” [49] as recommended by the authors.

CARDfree requires marker genes as the input in addition to the ST data. In all the analyses, the same gene markers used in SMART were used as the input for CARDfree. The inferred cell types, however, were not annotated with any cell type names. The same gene set enrichment analysis approach used in STdeconvolve was applied to label the cell types with cell type names.

The reference-based methods (RCTD, cell2location, spatialDWLS, CARD) were performed using the recommended settings according to the guidelines on their websites. These methods require a reference single-cell RNA-sequencing dataset as the input instead of a list of gene symbols used in marker-gene-assisted methods. More detailed information on running these methods can be found in Additional file 1: Supplementary Methods.

### Runtime evaluation

To benchmark the runtime of SMART, we randomly selected 50, 100, 200, 500, and 1000 spots from the simulated MPOA dataset. Using all 135 genes available in the dataset, the library size at each spot ranges from 3700 to 4000 gene counts. The runtime was measured using R package “tictoc” on a machine with an i7-4771 3.5 GHz CPU with 8 GB of RAM.

## Supplementary Information

Additional file 1: Supplementary Information. Supplementary Methods and Supplementary Figures.

Additional file 2: Table S1. Marker genes used in SMART for deconvolution of the MK dataset.

Additional file 3: Table S2. Marker genes used in SMART for deconvolution of the NSCLC dataset.

Additional file 4: Table S3. Marker genes used in SMART for deconvolution of the MPOA dataset.

Additional file 5: Table S4. Top 10 SMART-predicted cell type-specific genes in the MK dataset using different sets of marker genes and their overlap with marker genes.

Additional file 6. Review history.

## Data Availability

SMART is available as an R package under a GNU General Public License v3.0 license on GitHub [50] and Zenodo [51]. Data from the MK dataset [19] are available for download from https://figshare.com/projects/MERFISH_mouse_comparison_study/134213 [52]. Data from the MPOA dataset [26] are available at: https://datadryad.org/stash/dataset/doi:10.5061/dryad.8t8s248 [53]. The 10X Visium coronal section of the mouse brain dataset [27] is available for download at https://www.10xgenomics.com/resources/datasets/mouse-brain-section-coronal-1-standard-1-1-0. The PDAC data were obtained from sample A of the PDAC dataset [35] and are available at the Gene Expression Omnibus (accession number GSE111672) [54]. The simulated datasets used in this study are available at Zenodo [51].
