# Supplementary material for: SMART: spatial transcriptomics deconvolution using marker-gene-assisted topic model
Source: Genome Biol. 2024 Dec 2;25:304. doi: 10.1186/s13059-024-03441-1 (PMC11610197; doi:10.1186/s13059-024-03441-1)
Supplement: Supplementary file 1 — Additional file 1: Supplementary Information. Supplementary Methods and Supplementary Figures. [file 13059_2024_3441_MOESM1_ESM.docx]

**Supplementary Methods**

**Deconvolution using existing methods**

**RCTD**

RCTD was performed according to its online tutorial using the “full” mode.

nUMI <- colSums(STcounts)

puck <- SpatialRNA(coords, STcounts, nUMI)

barcodes <- colnames(puck@STcounts)

cell_types <- annot$free_annotation

reference <- Reference(STcounts, cell_types, nUMI)

myRCTD <- create.RCTD(puck, reference, max_cores = 1)

myRCTD <- run.RCTD(myRCTD, doublet_mode = 'full')

weights <- myRCTD@results$weights

norm_weights <- normalize_weights(weights)

**Cell2location**

Cell2location was performed according to its online tutorial at <https://cell2location.readthedocs.io/en/latest/notebooks/cell2location_tutorial.html>. The maximum training epochs was determined based on the resulting elbow plot. The ground truth mean number of cells per spot was used for the cell abundance parameter “N_cells_per_location”.

**Spatial DWLS**

SpatialDWLS was performed according to its online tutorial (<https://giottosuite.readthedocs.io/en/latest/subsections/datasets/mouse_visium_brain.html#dwls-spatial-deconvolution>). Leiden clustering was performed on the scRNA-seq reference dataset and marker genes were identified using R package “scran”. The top 30 marker genes were used to create the signature gene matrix for spatialDWLS.

giotto_SC <- createGiottoObject(

expression = t(counts),

instructions = instrs

)

giotto_SC <- addCellMetadata(giotto_SC,

new_metadata = annot.table)

giotto_SC<- normalizeGiotto(giotto_SC)

markers_scran <- findMarkers_one_vs_all(gobject=giotto_SC,

method="scran",

expression_values="normalized",

cluster_column = "Cell_class",

min_feats=3)

top_markers <- markers_scran[, head(.SD, 30), by="cluster"]

DWLS_matrix <- makeSignMatrixDWLSfromMatrix(matrix =

get_expression_values(giotto_SC,values = "normalized", output = "matrix"),

cell_type = pDataDT(giotto_SC)$Cell_class,

sign_gene = top_markers$feats)

goobj <- runDWLSDeconv(gobject = goobj,

sign_matrix = DWLS_matrix,

cluster_column = "leiden_clus")

**CARD**

CARD was performed with a minimum count of 100 per gene and a minimum count of 5 per spot.

CARD_obj = createCARDObject(

sc_count = sc_count,

sc_meta = sc_meta,

spatial_count = spatial_count,

spatial_location = spatial_location,

ct.varname = "cellType",

ct.select = unique(sc_meta$cellType),

sample.varname = 'sampleInfo',

minCountGene = 100,

minCountSpot = 5)

CARD_obj = CARD_deconvolution(CARD_object = CARD_obj)

**CARDfree**

CARDfree was performed using the same settings as in CARD. The marker genes used in CARDfree were the same as in SMART.

CARDfree_obj = createCARDfreeObject(

markerList = markers,

spatial_count = spatial_count,

spatial_location = spatial_location,

minCountGene = 100,

minCountSpot =5)

CARDfree_obj = CARD_refFree(CARDfree_obj)

**STdeconvolve**

STdeconvolve was performed according to its online tutorial. The number of latent topics “Ks” was chosen to match the number of cell types involved in the simulated datasets.

ldas <- fitLDA(as.matrix(ST_dat), Ks = 8,seed=1)

optLDA <- optimalModel(models = ldas, opt = "min")

results <- getBetaTheta(optLDA, perc.filt = 0.05, betaScale = 1000)

deconProp <- results$theta

deconGexp <- results$beta

**Celloscope**

The following default parameters were used for Celloscope according to its online tutorial. The marker genes used in Celloscope were the same as in SMART. The ground truth number of cells per spot was used for cell abundance.

{"number of iterations": 15000, "burn in": 10000,

"mode number of cells": "ASPRIORS",

"a":10, "b":1, "a_0":0.1, "b_0":1,

"alpha": 8,

"step size thetas": 0.1,

"step size number of cells": 2.01,

"step size lambda_0": 0.05,

"step size p_g": 0.1,

"thinning_parameter": 10,

"number of cells prior strength": 2,

"how often update step size": 3000,

"how often drop": 10}

**The sampling algorithm in SMART**

SMART builds upon keyATM and follows the sampling algorithm in keyATM. Suppose there are $V$ genes and $D$ spots and the set of marker genes is represented by $\mathcal{V}_{k}$ for each cell type $k$ with marker genes. The sampling distribution of cell type assignment for each mRNA molecule $i$ in spot $d$ is given by

$$\Pr\left( z_{di}=k | \mathbf{z}^{-di}, \mathbf{w}, \mathbf{s}, \boldsymbol{\alpha}, \boldsymbol{\beta}, \tilde{\boldsymbol{\beta}}, \boldsymbol{\gamma} \right)\propto\left\{ \begin{aligned} \frac{\beta_{v}+n_{kv}^{-di}}{\Sigma_{v} \beta_{v}+n_{k}^{-di}}\cdot\frac{n_{k}^{-di}+\gamma_{2}}{\tilde{n}_{k}^{-di}+\gamma_{1}+n_{k}^{-di}+\gamma_{2}}\cdot(n_{dk}^{-di}+\alpha_{k})\text{ if }s_{di}=0, \\ \frac{\tilde{\beta}_{v}+\tilde{n}_{kv}^{-di}}{\Sigma_{v\in\mathcal{V}_{k}} \tilde{\beta}_{v}+\tilde{n}_{k}^{-di}}\cdot\frac{\tilde{n}_{k}^{-di}+\gamma_{1}}{\tilde{n}_{k}^{-di}+\gamma_{1}+n_{k}^{-di}+\gamma_{2}}\cdot\left( n_{dk}^{-di}+\alpha_{k} \right)\text{ if }s_{di}=1, \end{aligned} \right.$$

where $n_{k}^{-di}$( $\tilde{n}_{k}^{-di}$) represents the number of mRNA molecules (marker gene mRNA molecules) in the spots assigned to cell type $k$, excluding the $i$th molecule of spot $d$. $n_{kv}^{-di}$($\tilde{n}_{kv}^{-di}$) represents the number of times gene (marker gene) $v$ is assigned to cell type $k$, excluding the $i$th molecule of spot $d$. $n_{dk}^{-di}$ represents the number of times gene $v$ is assigned to cell type $k$ in spot $d$, excluding the $i$th molecule of spot $d$.

The conditional posterior distribution for $s_{di}$ is given by

$$Pr(s_{di}=s|\mathbf{s}^{-di},\boldsymbol{z} \mathbf{w}, \boldsymbol{\beta}, \tilde{\boldsymbol{\beta}}, \boldsymbol{\gamma})\propto\left\{ \begin{aligned} \frac{\beta_{v}+n_{z_{di}v}^{-di}}{\Sigma_{v} \beta_{v}+n_{z_{di}}^{-di}}\cdot(n_{z_{di}}^{-di}+\gamma_{2})\text{ if }s=0. \\ \frac{\tilde{\beta}_{v}+\tilde{n}_{z_{di}v}^{-di}}{\Sigma_{v\in\mathcal{V}_{z_{di}}} \tilde{\beta}_{v}+\tilde{n}_{z_{di}}^{-di}}\cdot\left( \tilde{n}_{z_{di}}^{-di}+\gamma_{1} \right)\text{ if }s=1. \end{aligned} \right.$$

The conditional posterior distribution of $\alpha_{k}$ is given by

$$p\left( \alpha_{k} | \boldsymbol{\alpha}_{-[k]}, \mathbf{s}, \mathbf{z},\mathbf{w}, \tilde{\boldsymbol{\eta}} \right)\propto\frac{\Gamma\left( \Sigma_{k=1}^{K} \alpha_{k} \right)\Pi_{d=1}^{D}\Gamma\left( n_{dk}+\alpha_{k} \right)}{\Gamma\left( \alpha_{k} \right)\Pi_{d=1}^{D}\Gamma\left( \Sigma_{k=1}^{K} n_{dk}+\alpha_{k} \right)}\cdot\alpha_{k}^{\tilde{\eta}_{1}-1}\exp\left( -\tilde{\eta}_{2}\alpha_{k} \right),$$

for cell type $k=1, 2, \ldots, \tilde{K}$. For a “no-marker” cell type $k=\tilde{K}+1, \ldots, K$, $\tilde{\eta}_{1}$and $\tilde{\eta}_{2}$ are replaced with $\eta_{1}$ and $\eta_{2}$.

For a cell type $k$ with marker genes, the final gene frequency distribution $\phi_{k}^{*}$ is a mixture of $\phi_{k}$ and $\tilde{\phi}_{k}$, given by

$$\phi_{kv}^{*}=\left( 1-\pi_{k} \right)\phi_{kv}+\pi_{k}\tilde{\phi}_{kv}$$

The marginal posterior mean of the gene frequency distribution is given by

$$\mathbb{E}\left[ \phi_{kv}^{*} | \mathbf{w} \right]\mathbb{=E}\left\{ \mathbb{E}\left[ \phi_{kv}^{*} | \beta_{v},\tilde{\beta}_{v},\boldsymbol{\gamma},\mathbf{s},\boldsymbol{z},\mathbf{w} \right] \right.\left| \mathbf{w} \right\}=\left\{ \begin{aligned} \mathbb{E}\left[ \frac{n_{k}+\gamma_{2}}{\tilde{n}_{k}+\gamma_{1}+n_{k}+\gamma_{2}}\cdot\frac{\beta_{v}+n_{kv}}{\Sigma_{v^{'}} \beta_{v^{'}}+n_{k}}+\frac{\tilde{n}_{k}+\gamma_{1}}{\tilde{n}_{k}+\gamma_{1}+n_{k}+\gamma_{2}}\cdot\frac{\tilde{\beta}_{v}+\tilde{n}_{kv}}{\Sigma_{v^{'}\in V_{k}}\tilde{\beta}_{v^{'}}+\tilde{n}_{k}} | \mathbf{w} \right]\text{ if }v\in\mathcal{V}_{k}, \\ \mathbb{E}\left[ \frac{n_{k}+\gamma_{2}}{\tilde{n}_{k}+\gamma_{1}+n_{k}+\gamma_{2}}\cdot\frac{\beta_{v}+n_{kv}}{\Sigma_{v^{'}} \beta_{v^{'}}+n_{k}} | \mathbf{w} \right]\text{ if }v\notin\mathcal{V}_{k}, \end{aligned} \right.$$

where $n_{k}=\Sigma_{v=1}^{V} n_{kv}$, and $\tilde{n}_{k}=\Sigma_{v=1}^{V} \tilde{n}_{kv}$.

The marginal posterior of $\theta_{dk}$ for each spot $d$ and cell type $k$ is given by,

$$\mathbb{E}\left[ \theta_{dk} | \mathbf{w} \right]\mathbb{=E\{E}\left[ \theta_{dk} | \alpha_{k},\boldsymbol{z},\mathbf{w} \right]\left| \mathbf{w} \right\}\mathbb{=E}\left[ \frac{\alpha_{k}+n_{dk}}{\Sigma_{k^{'}=1}^{K}\alpha_{k^{'}}+n_{dk^{'}}} | \mathbf{w} \right]$$

We recommend consulting the original paper of keyATM for additional details.

**Supplementary Figures**

**
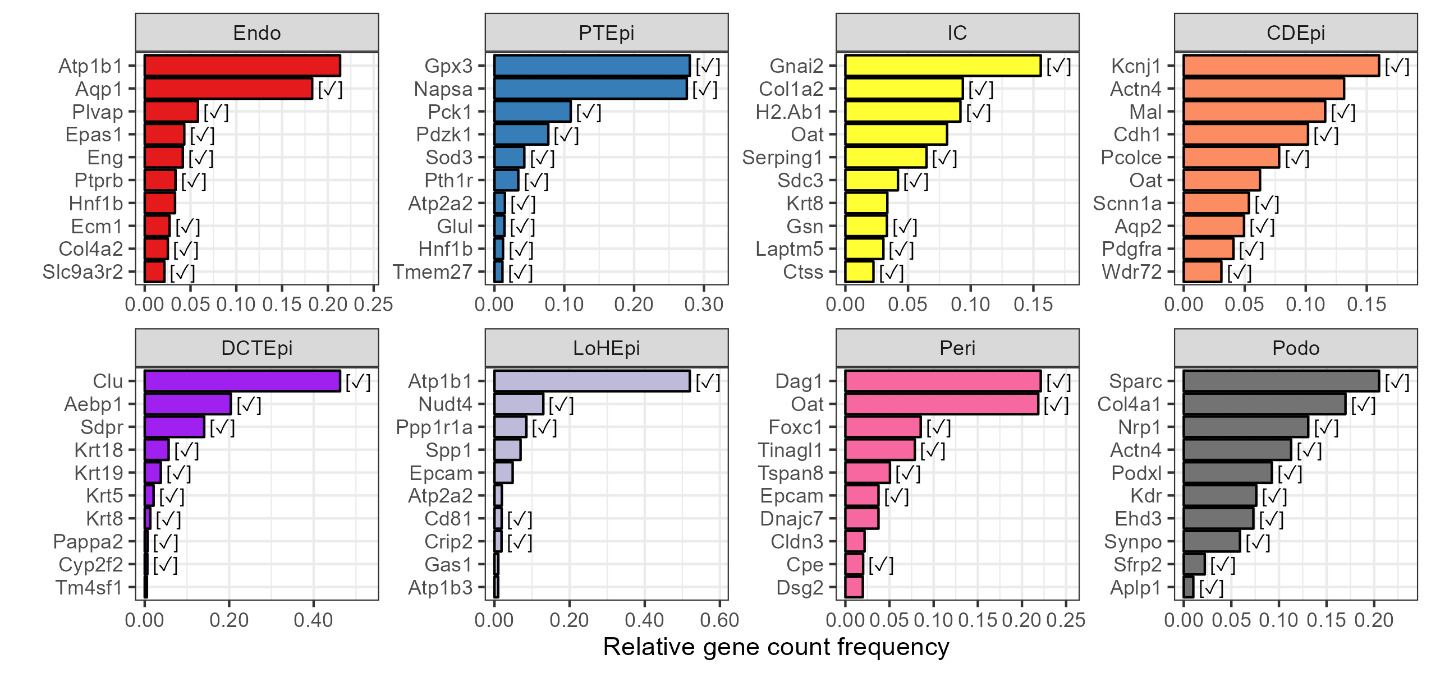
**

**Fig. S1 |** Top 10 SMART-predicted cell type-specific genes in the MK simulated dataset based on the GT marker genes. Genes that were used as the marker genes for SMART deconvolution were labeled with checkmarks. Abbreviations: endothelial cell (Endo), epithelial cell of the proximal tubule (PTEpi), immune cell (IC), collecting duct epithelial cell(CDEpi), distal convoluted tubule epithelial cell (DCTEpi), loop of Henle epithelial cell (LoHEpi), pericyte (Peri), podocyte (Podo).


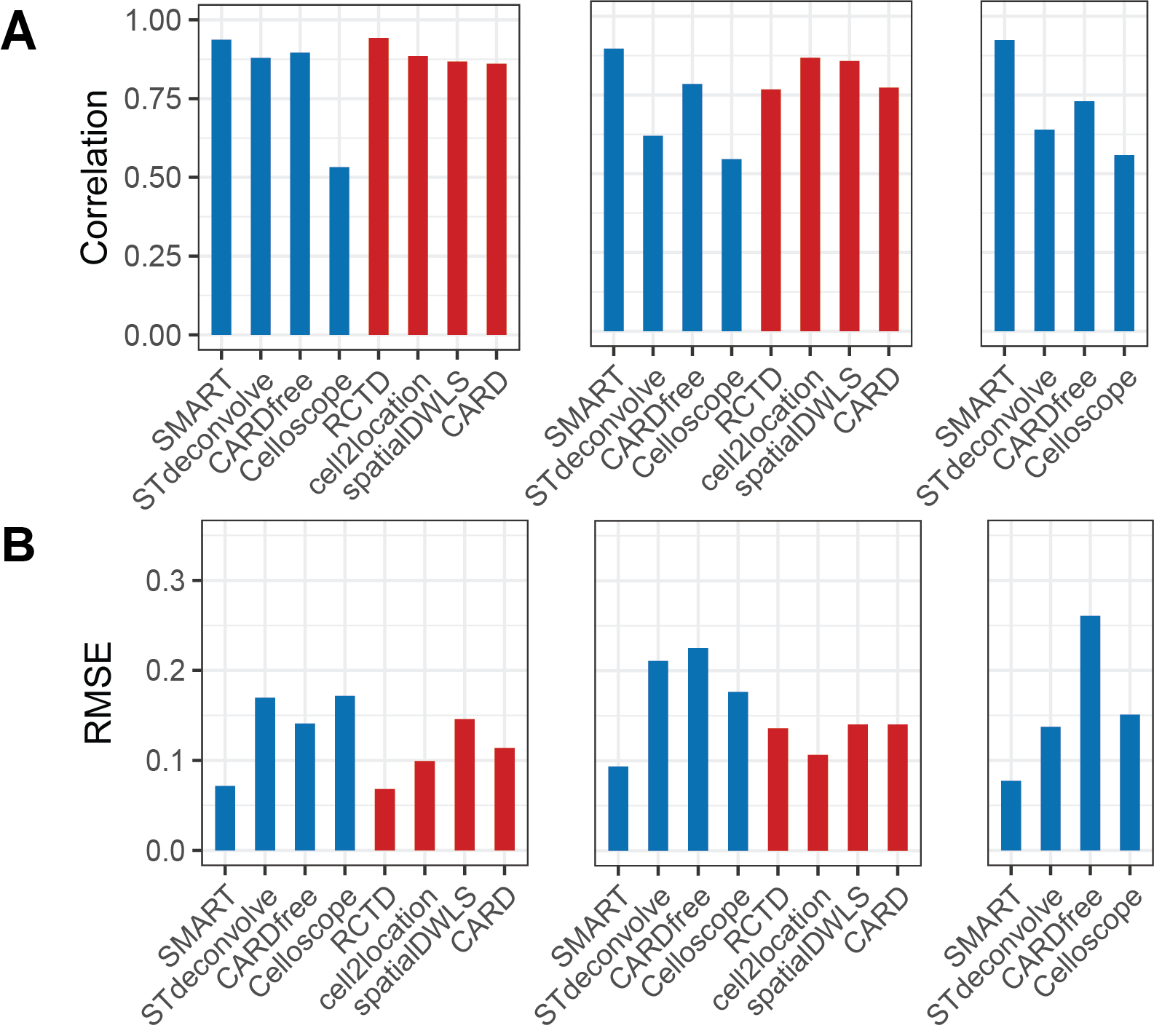


**Fig. S2 |** The Pearson correlation (**A**) and the RMSE (**B**) between the predicted and the GT cell type proportions across all spots using the GT markers/reference (left), the TMS markers/reference (middle), and the literature-based markers (right) in the MK dataset. Blue = marker-assisted/reference-free methods; Red = reference-based methods.


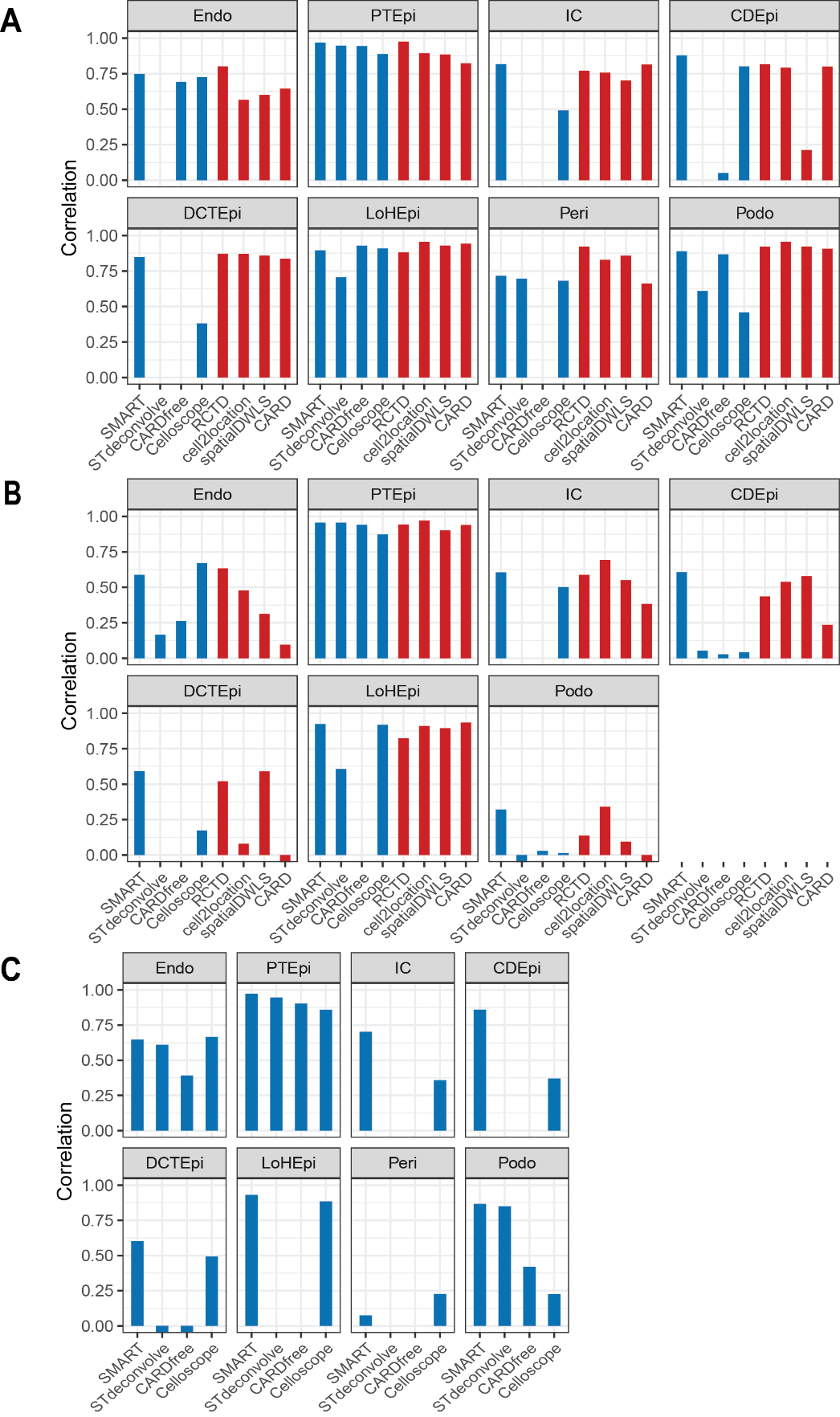


**Fig. S3 |** The Pearson correlation between the predicted and the GT cell type proportions in each cell type using the GT markers/reference (**A**), the TMS markers/reference (**B**), and the literature-based markers (**C**) in the MK dataset. For STdeconvolve and CARDfree, no bar is shown for a certain cell type if the method did not successfully identify the cell type. Blue = marker-assisted/reference-free methods; Red = reference-based methods.

**
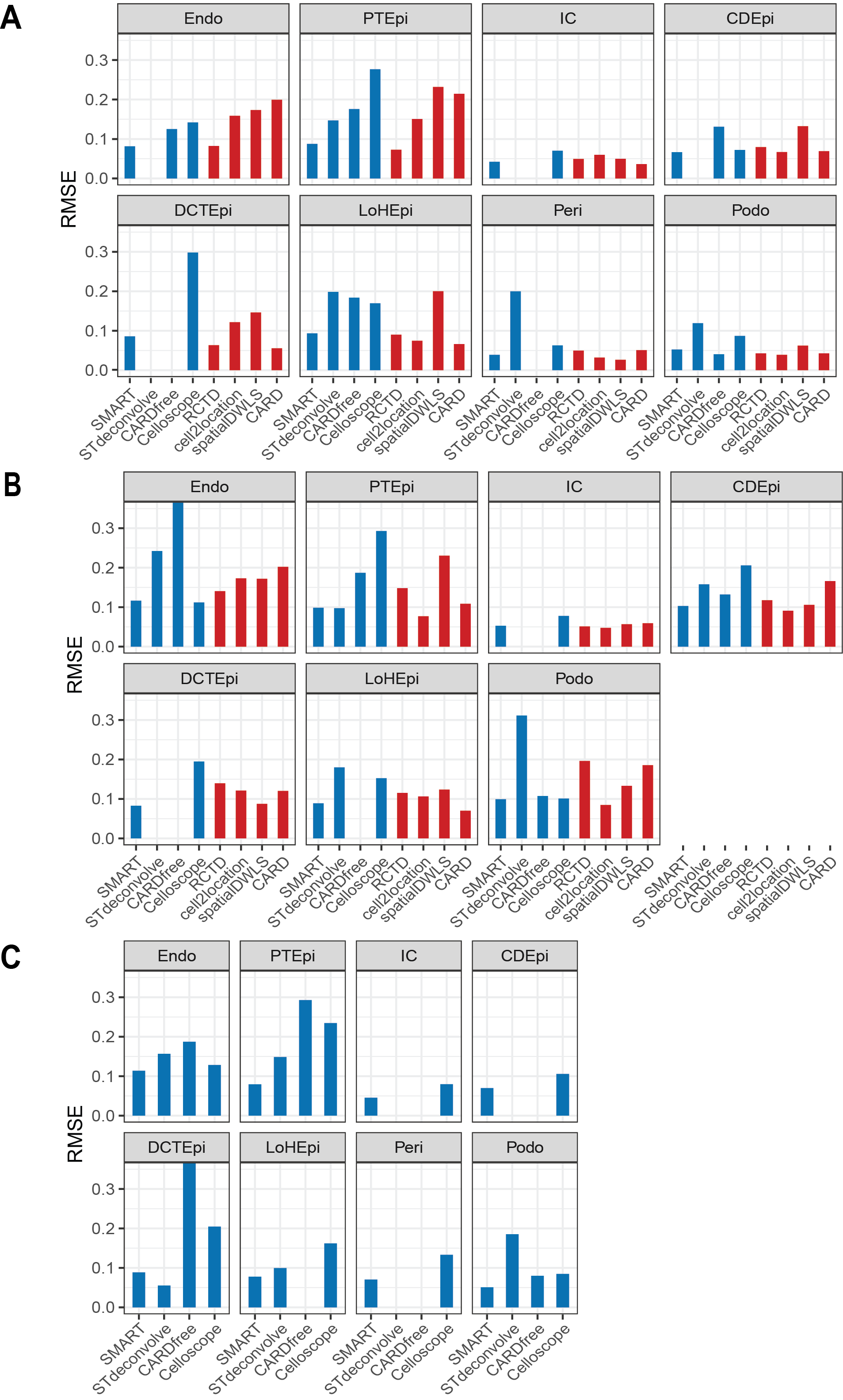
**

**Fig. S4 |** The overall RMSE between the predicted and the GT cell type proportions in each cell type using the GT markers/reference (**A**), the TMS markers/reference (**B**), and the literature-based markers (**C**) in the MK dataset. For STdeconvolve and CARDfree, no bar is shown for a certain cell type if the method did not successfully identify the cell type. Blue = marker-assisted/reference-free methods; Red = reference-based methods.


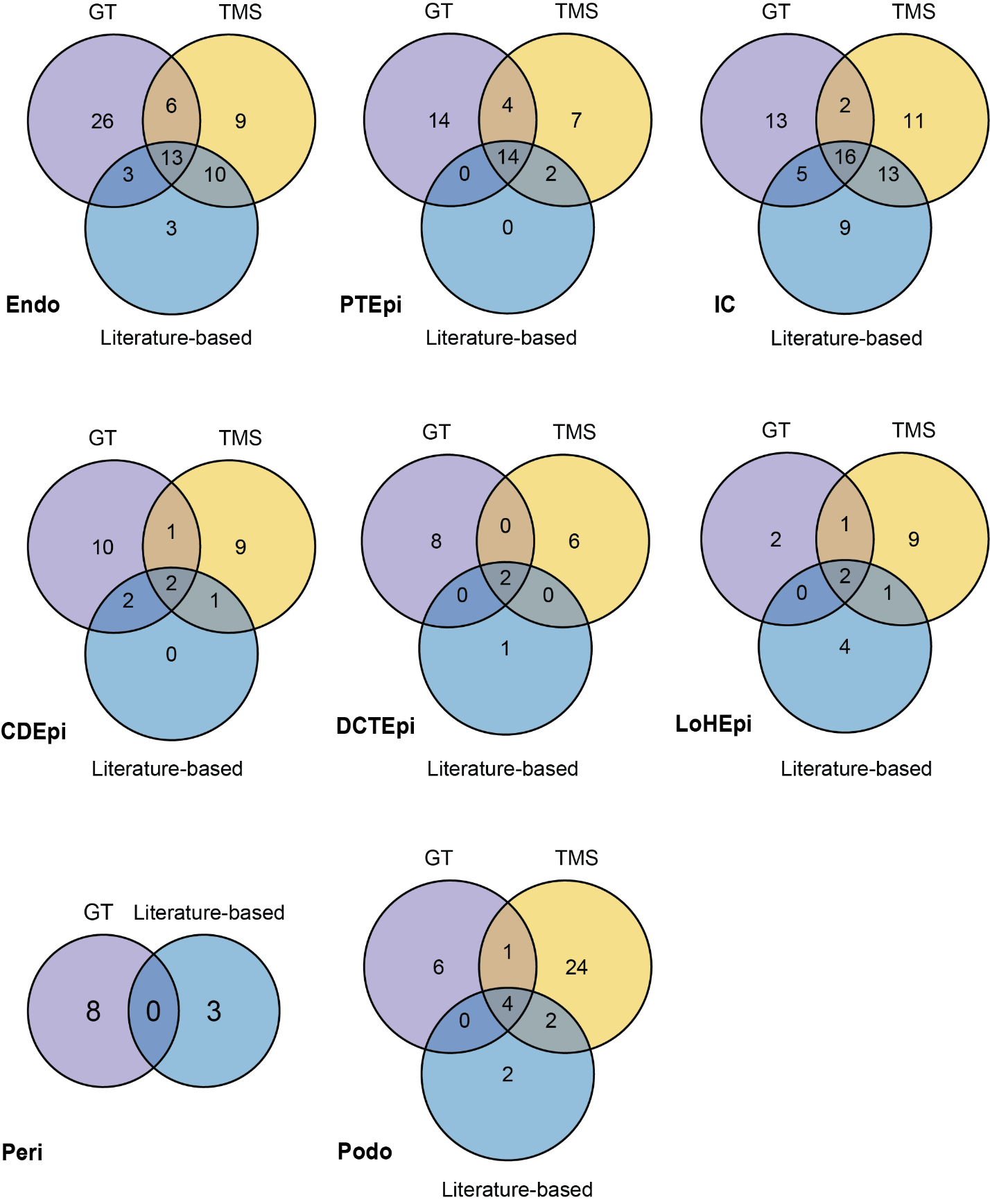


**Fig. S5 |** Venn diagrams showing the number of overlapping genes among the GT, the TMS, and the literature-based marker genes used for SMART deconvolution of the simulated MK dataset. Abbreviations: endothelial cell (Endo), epithelial cell of the proximal tubule (PTEpi), immune cell (IC), collecting duct epithelial cell(CDEpi), distal convoluted tubule epithelial cell (DCTEpi), loop of Henle epithelial cell (LoHEpi), pericyte (Peri), podocyte (Podo).

**
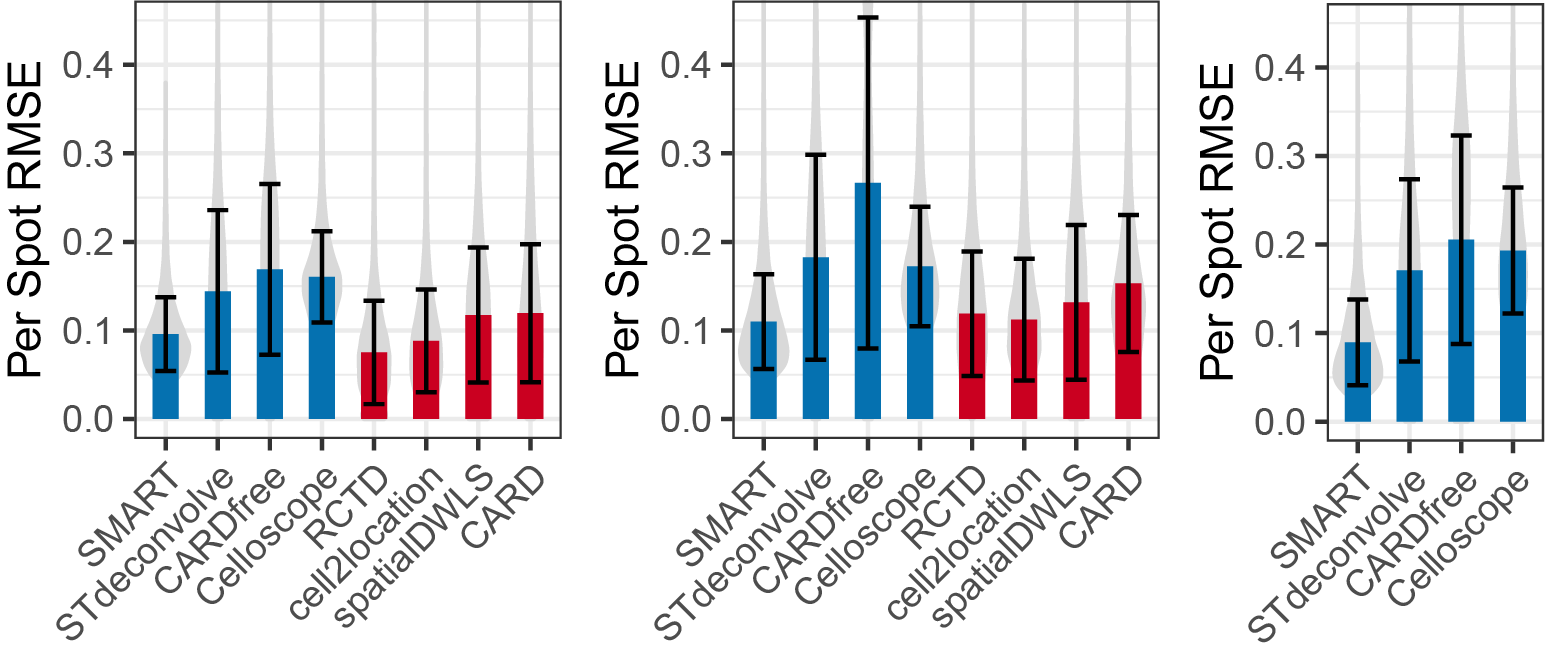
**

**Fig. S6 |** The per-spot RMSE between the predicted and the GT cell type proportions using the GT markers/reference (left), the TMS markers/reference (middle), and the literature-based markers (right) in the re-simulated MK dataset with, on average, 10 cells per spot. Blue = marker-assisted/reference-free methods; Red = reference-based methods.

**
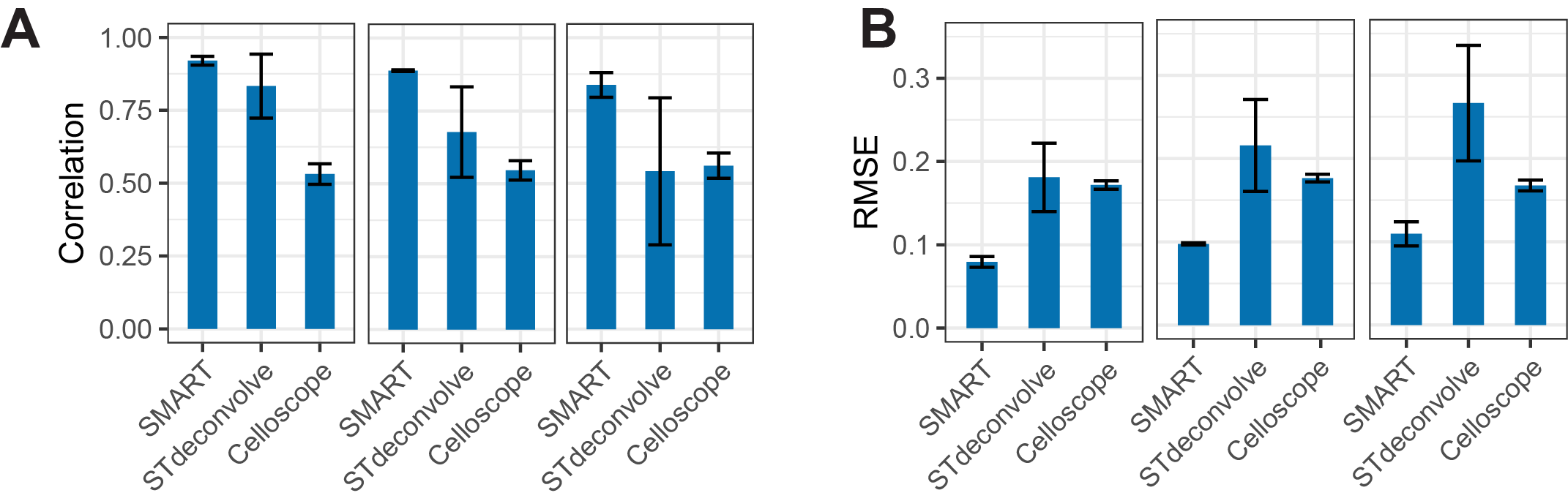
**

**Fig. S7 |** Between-run variability of SMART, STdeconvolve, and Celloscope. (**A**) The Pearson correlation between the predicted and the GT cell type proportions across all spots over 100 repeats using the GT markers (left), the TMS markers/reference (middle), and the literature-based markers (right). (**B**) The RMSE between the predicted and the GT cell type proportions across all spots over 100 repeats using the GT markers (left), the TMS markers/reference (middle), and the literature-based markers (right). The error bars represent mean ± SD.

**
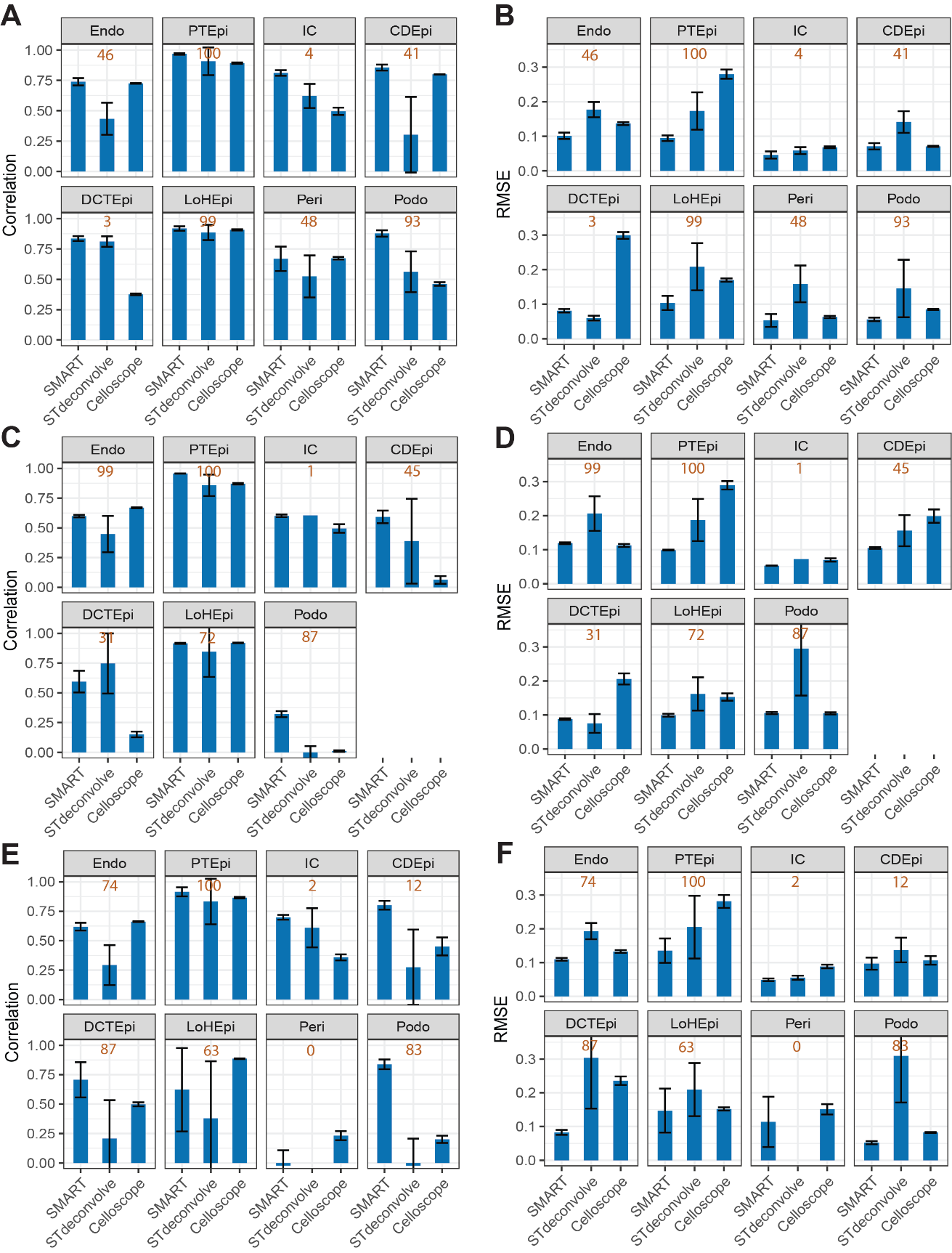
**

**Fig. S8 |** Between-run variability of SMART, STdeconvolve, and Celloscope for each cell type. The Pearson correlation and RMSE between the predicted and the GT cell type proportions for each cell type over 100 repeats using the GT markers (**A, B**), the TMS markers/reference (**C, D**), and the literature-based markers (**E, F**). The error bars represent mean ± SD. Since STdeconvolve did not identify all cell types at each repeated run, the number of repeated runs identifying a certain cell type in STdeconvolve is indicated in **A-F**. SMART and Celloscope identified all cell types in all 100 repeated runs.

**
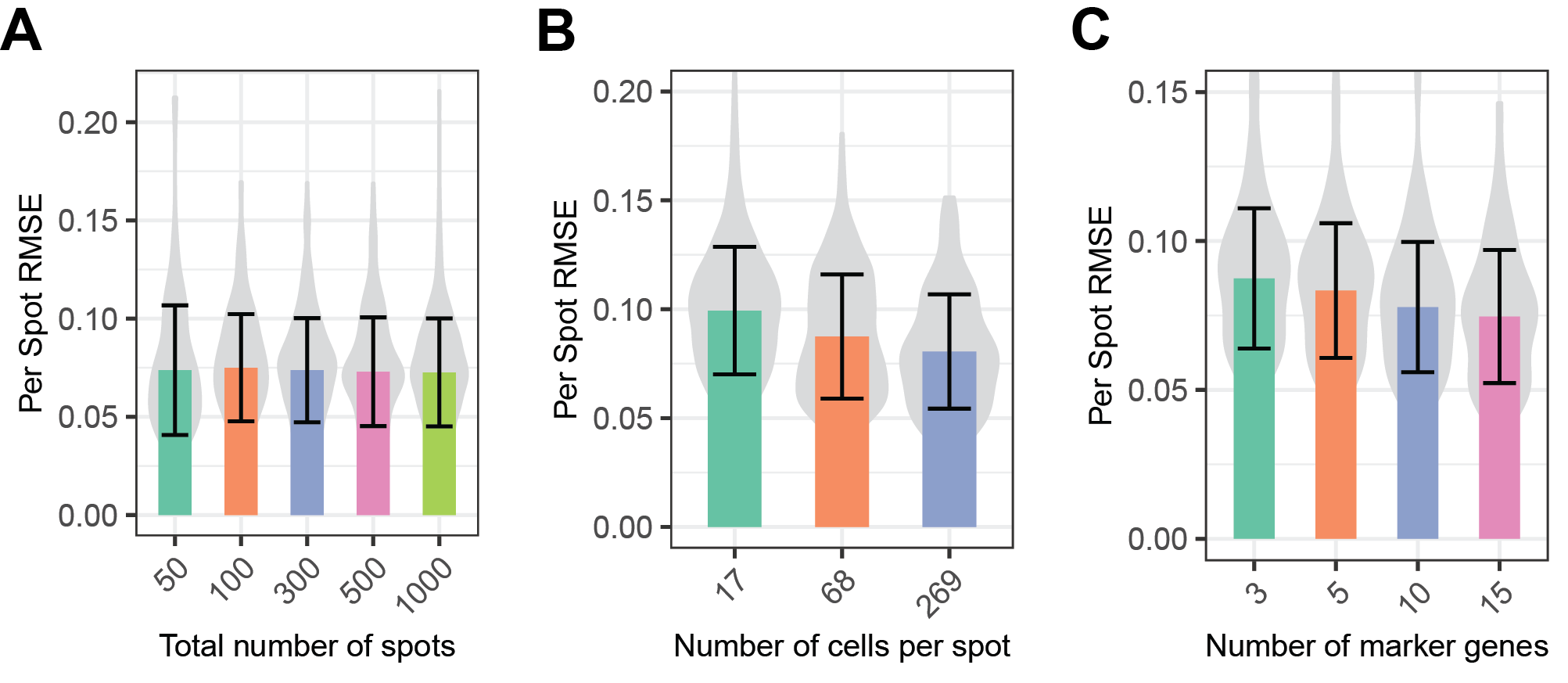
 Fig. S9 |** Factors that may affect the performance of SMART. (A) The number of total spots. (B) The number of cells on average within each spot. (C) The number of marker genes. The error bars represent mean ± SD.

**
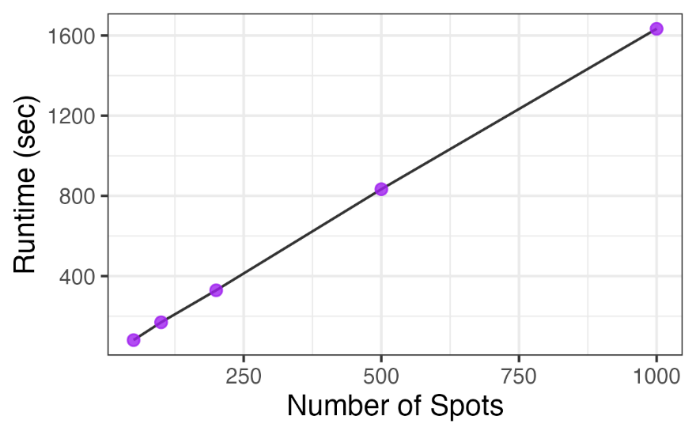
**

**Fig. S10 |** Benchmarking the runtime of SMART using the MPOA simulated dataset.


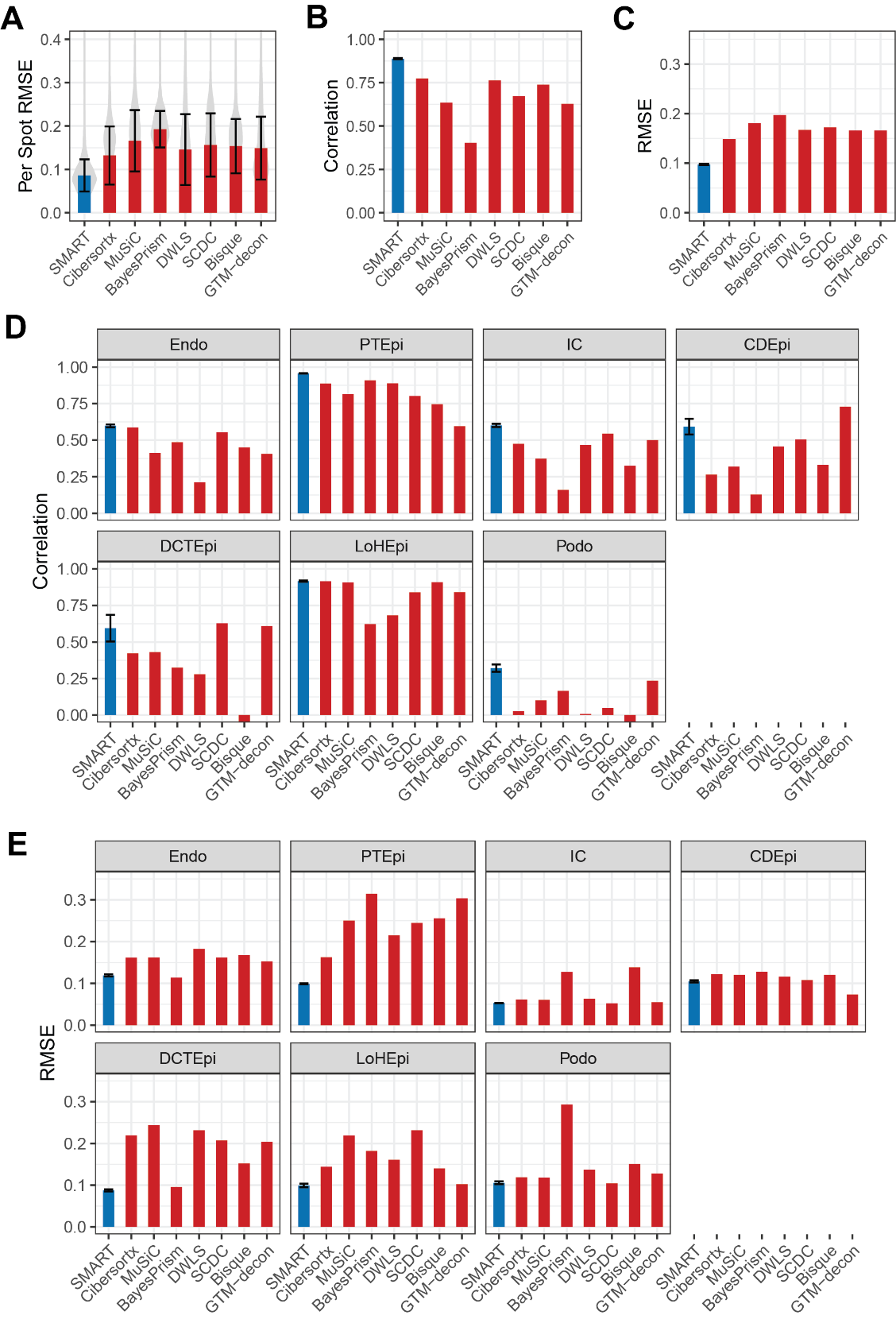


**Fig. S11 |** Performance evaluation of SMART and bulk transcriptomics deconvolution methods using simulated ST data in mouse kidney. With the TMS markers/reference, SMART demonstrated lower per-spot RMSE (**A**), stronger correlations (**B**), and lower RMSE (**C**) between the predicted and the GT cell type proportions and outperformed the bulk transcriptomics deconvolution methods. SMART also demonstrated stronger correlations (**D**) and lower RMSE (**E**) between the predicted and the GT cell type proportions in each cell type. Blue = marker-assisted ST deconvolution method (SMART); Red = reference-based bulk transcriptomics deconvolution methods (Cibersortx, MuSiC, BayesPrism, DWLS, SCDC, Bisque, and GTM-decon).
