# Supplementary material for: SMART: spatial transcriptomics deconvolution using marker-gene-assisted topic model
Source: Genome Biol. 2024 Dec 2;25:304. doi: 10.1186/s13059-024-03441-1 (PMC11610197; doi:10.1186/s13059-024-03441-1)
Supplement: Supplementary file 6 — Additional file 6. Review history. [file 13059_2024_3441_MOESM6_ESM.docx]

Review History

**First round of review**

**Reviewer 1**

Yes: Statistics and statistical tests appear to be fine

**Were you able to assess all statistics in the manuscript, including the appropriateness of statistical tests used?**

**Were you able to directly test the methods?**

Yes

**Comments to author:**

Many spatial transcriptomics (ST) technologies lack single-cell resolution. Using computational algorithms as an auxiliary to decipher the cell type decomposition within each spatial spot will facilitate downstream analysis. To address this limitation, Yang et al. developed SMART, a deconvolution method that utilized cell type-specific gene expression profiles to determine the cellular compositions of ST data. The manuscript reveals an interesting methodology for ST analysis, and most of the results are well presented. However, the reviewer has the following concerns:

1. The authors used the term "reference-free" to describe their model. However, they still utilized single-cell reference data to extract the marker genes for each cell type in their examples. Due to batch effects, the selected marker genes vary between different reference datasets, which will further affect the performance of deconvolution. Specifically, in benchmark comparisons, the authors used the single cells directly from the MERFISH data to compute cell type-specific differentially expressed genes as marker genes. This does not truly represent a "reference-free" approach. Instead, using words such as "marker-gene driven" would provide a more precise description of their model. In a "reference-free" model, one of its superiority lies in its ability to correct batch effects, which has also mentioned in the "Introduction" section ([Page 3, line 2] "in some methods, the batch effects between the target ST dataset and the reference profile are not properly handled, resulting in inaccurate results"). Therefore, the authors should consider utilizing uniformed gene markers from a third party such as CellMarker database (PMID: 30289549), instead of extracting gene markers extracted from reference datasets to evaluate their model.
2. A similar study by Celloscope [PMID: 37198601] also employed cell type marker genes for cell type deconvolution. I think the authors should also include this model in their comparison.
3. STdeconvolve does not require any prior cell type information for conducting cell type deconvolution. Therefore, it is unfair to compare SMART with STdeconvolve in Figure 2E and 2F. A more appropriate comparison would involve considering Celloscope mentioned in comment 2 and CARDfree.
4. The authors used a single slice from a MERFISH dataset for benchmarking. However, using only one slice is not sufficient to adequately demonstrate the advantages of SMART across diverse datasets. Additionally, the selected MERFISH dataset encompasses only 304 genes. To provide a more comprehensive evaluation, the authors should consider including a genome-wide ST dataset, such as slide-seq, as a complementary benchmark.
5. For platform such as 10X Visium, the cell number of a spot can be as low as 1~10. However, in Figure 1, most simulated spots contain much more cells. I think the authors should also benchmark these models on spots with fewer cells.
6. The example of "two-stage approach" is not convincing to support the "reference-free" ability of their model. In the manuscript, the authors have priori knowledge of three T subtypes and two DC subtypes in this dataset, and they used these reference single cells to extract marker genes to process their model. However, it's important to note that reference-free models are usually considered in a situation where an ideal reference dataset is unavailable. In real-world data, we usually lack priori subtype information for a ST dataset or an idea reference dataset containing a hierarchical cell type annotation. Therefore, a one-stage deconvolution is more realistic for most real-world scenarios.
7. In terms of SMART, it can also predict cell type-specific gene expression as part of the output. However, I did not find any assessment of the performance of this aspect. The authors should also evaluate the accuracy of gene expression prediction results. Particularly, assessing its performance in predicting cell counts in genome-wide data is important for users.
8. Figure 1 should be improved. The workflow within the SMART model box is too simple and will cause confusion to readers. The authors should emphasize the key components of the model intuitively. In addition, the authors should provide the annotations for these alphabet labels.
9. Since the heatmaps in Figure 2C, 3C and 4C show the Pearson correlation coefficients between the predicted and the GT cell type proportions for each cell type, why are these matrices asymmetrical?
10. Figure 4 line 4: "(E)" should be "(D)".

**Reviewer 2**

**Were you able to assess all statistics in the manuscript, including the appropriateness of statistical tests used?**

No

**Were you able to directly test the methods?**

No

**Comments to author:**

Authors propose a topic model called SMART, which is closely based off the existing algorithm called keyATM to deconvolve spot from ST data. The overall writing is clear but the paper lacks technical contribution and misses some important intuition and analyses.

Major comments:
1. One major limitation of this approach is that it does not make use of the spatial information, which makes it a similar approach as other non-ST deconvolution methods.
2. Since the proposed model does not use spatial info, how does it compare with well-known and recently developed non-spatial deconvolution method like Cibersort, MuSiC, BayesPrism, and GTM-decon. Latter two are reference-based guided topic model and able to deconvolve bulk transcriptome including ST spots.
3. The authors wrote "if the variable equals 0, 𝑤_di is drawn from the gene frequencies for cell types without marker genes 𝜙_k". This makes no sense. If phi_k is ONE topic distribution over all genes except marker genes for cell type k, how can it be "the gene frequencies for cell types without marker genes" (note here cell types are plural)?
4. In NLP, s_di=1 means that the keyword w_di is sampled from the key topic k whose distribution is over the keywords; s_di=0 means that the keyword is sampled from a standard topic, whose distribution is over the entire vocabulary. When s_di = 0, does that mean the cell is not cell type k but somehow expresses the marker gene?
5. Although the algorithm closely follows keyATM, sufficient details should still be provided for the Gibbs sampling algorithm.
6. To see how well each cell type being deconvolved, authors should evaluate the correlation per cell type across cells rather than correlation across all cells and cell types.
7. A main benefits of topic model is the interpretable topic distribution over genes. Yet no topic analysis was performed in the paper. For example, what are the top genes for phi_k and phi_tilde for each cell type k?
8. I agree that having overlapping marker genes between cell types can lead to poor performance of the keyATM algorithm. However, the proposed two-stage approach is ad hoc and feels arbitrary.

Minor comments:
1. Fig. 2: Having the groundtruth composition on the side is helpful for comparison.
2. Fig. 4 caption panel E should have been panel 'D'

**Authors Response**

**Point-by-point responses to the reviewers’ comments:**

Reviewer reports:

Reviewer #1: Many spatial transcriptomics (ST) technologies lack single-cell resolution. Using computational algorithms as an auxiliary to decipher the cell type decomposition within each spatial spot will facilitate downstream analysis. To address this limitation, Yang et al. developed SMART, a deconvolution method that utilized cell type-specific gene expression profiles to determine the cellular compositions of ST data. The manuscript reveals an interesting methodology for ST analysis, and most of the results are well presented. However, the reviewer has the following concerns:

1. The authors used the term "reference-free" to describe their model. However, they still utilized single-cell reference data to extract the marker genes for each cell type in their examples. Due to batch effects, the selected marker genes vary between different reference datasets, which will further affect the performance of deconvolution. Specifically, in benchmark comparisons, the authors used the single cells directly from the MERFISH data to compute cell type-specific differentially expressed genes as marker genes. This does not truly represent a "reference-free" approach. Instead, using words such as "marker-gene driven" would provide a more precise description of their model. In a "reference-free" model, one of its superiority lies in its ability to correct batch effects, which has also mentioned in the "Introduction" section ([Page 3, line 2] "in some methods, the batch effects between the target ST dataset and the reference profile are not properly handled,
resulting in inaccurate results"). Therefore, the authors should consider utilizing uniformed gene markers from a third party such as CellMarker database (PMID: 30289549), instead of extracting gene markers extracted from reference datasets to evaluate their model.

We thank the reviewer for the suggestion. In the benchmarking example, we provided two scenarios: 1) an ideal scenario where we use the MERFISH data itself for reference-based approaches and use the markers directly derived from the MERFISH data for reference-free approaches. This ideal scenario minimizes any confounding factors that may affect the benchmarking results and provides the best possible results for both the reference-based and reference-free methods. 2) The second scenario is a more realistic scenario where we take an external dataset (the TMS dataset) as the reference for reference-based approaches and use the markers derived from the external dataset for reference-free approaches. As is often the case, it is difficult for researchers to find a reference dataset with the same experimental and technical details.

In both scenarios, for reference-free methods, we used markers derived from the original dataset or the TMS dataset to make the results between reference-based methods and reference-free methods more comparable and fairer because the reference datasets and the markers derived from them are transcriptionally similar.

To demonstrate the advantage of SMART as a reference-free method, as suggested, we added a third scenario where we used markers that we collected from the CellMarker 2.0 database and existing literature (Miao *et al.*). (See revised manuscript line 149-159)

Moreover, as suggested by the reviewer, we have changed the term “reference-free” to “marker-assisted” to describe our method. Although STdeconvolve is considered a reference-free method, it still uses marker genes and gene set enrichment analysis after deconvolution to identify the latent cell types with cell type names. Therefore, in the benchmarking analysis (Figure 2 and Supp Figure 2), we have included it in the “marker-assisted” category.

2. A similar study by Celloscope [PMID: 37198601] also employed cell type marker genes for cell type deconvolution. I think the authors should also include this model in their comparison.

We thank the reviewer for the suggestion. We have now included Celloscope in the main benchmarking analysis (Figure 2 and Supp Figure 2). Using the same marker genes and the default parameters, Celloscope was outperformed by SMART in all three scenarios. The variance in per spot RMSE is also higher in Celloscope compared to SMART (Figure 2G). (See revised manuscript line 98-184)

3. STdeconvolve does not require any prior cell type information for conducting cell type deconvolution. Therefore, it is unfair to compare SMART with STdeconvolve in Figure 2E and 2F. A more appropriate comparison would involve considering Celloscope mentioned in comment 2 and CARDfree.

The prediction accuracy of all the methods was compared in Figure 2. The purpose of this section (on page 8) was not to compare the prediction accuracy but to demonstrate that unsupervised methods such as STdeconvolve have high variability between runs (because of different starting values [random seeding]), but semi-supervised approaches such as SMART could be more stable and reproducible by including marker genes as prior knowledge. This is also one of our motivations for developing SMART because the results of STdeconvolve could be quite different if a different starting value is used, even when interrogating the same dataset. Again, although STdeconvolve does not need marker gene information for conducting cell type deconvolution, it still requires marker genes afterwards to label the latent cell types. We have re-worded this section to highlight the motivation for using semi-supervised approaches (SMART) over unsupervised methods (STdeconvolve). Moreover, as the reviewer suggested, we have added Celloscope to this section. Celloscope, as a semi-supervised approach, also demonstrated small variability similar to SMART; however, SMART shows better accuracy in terms of correlation and RMSE (Supplementary Figure 3 & Figure 2G). We have now moved the original Figure 2E and 2F to Supplementary Figure 3. (See revised manuscript line 161-184)

4. The authors used a single slice from a MERFISH dataset for benchmarking. However, using only one slice is not sufficient to adequately demonstrate the advantages of SMART across diverse datasets. Additionally, the selected MERFISH dataset encompasses only 304 genes. To provide a more comprehensive evaluation, the authors should consider including a genome-wide ST dataset, such as slide-seq, as a complementary benchmark.

We thank the reviewer for the suggestion. In Figure 4 where we showcase the covariate model, we have extended the simulated MPOA dataset to include 6 mice (3 female mice and 3 male mice) and in total 47 slices to demonstrate the applicability of SMART on multiple slices and multiple samples. Figure 4C shows a correlation of >0.7 between the predicted and ground truth cell type proportions in all cell types, indicating that SMART performs well across multiple samples. (See revised manuscript line 222-250)

To reduce the runtime of SMART, we recommend only using the marker genes and highly variable genes in a ST dataset. Therefore, even on a genome-wide ST dataset, we recommend doing a gene selection procedure and only taking a subset of the genes. We have added this as a note to line 310-312 of the manuscript. We skipped this gene selection procedure in the benchmarking analysis of the simulated datasets because there were only 304 genes. Although the benchmarking only contains 304 genes, we believe that it could be a good representation of a case where a gene selection procedure has already been performed. Additionally, Figure 5 shows the application of SMART on a genome-wide dataset (the 10X Visium mouse brain dataset). In this dataset, we have selected ~2900 genes with marker genes and high variability out of 29,000 genes. The predicted cell type proportions and spatial organization of the cells are consistent with the existing literature as expected. We have added a note to the main text to highlight that a gene selection procedure is recommended for ST datasets with more genes. (See revised manuscript line 253-275)

5. For platform such as 10X Visium, the cell number of a spot can be as low as 1~10. However, in Figure 1, most simulated spots contain much more cells. I think the authors should also benchmark these models on spots with fewer cells.

We thank the reviewer for the suggestion. Since platforms such as Nanostring GeoMx contain at least 50 cells in each spot, in the main benchmarking analysis (Figure 2), we simulated the spots to contain 51 cells/spot on average. As a sensitivity analysis, we have re-simulated the spots to contain on average 10 cells/spot and repeated the benchmarking (Supp Fig 2). The results were similar to Figure 2G except that the performance of cell2location became slightly better than SMART when an ideal reference was used (Supp Fig 2 left). SMART still shows the best performance in realistic scenarios where an external set of markers (Supp Fig 2 middle) or a set of markers collected from existing literature and databases (Supp Fig 2 right) was used. These results indicate that SMART is robust for both high-resolution platforms and lower-resolution platforms. (See revised manuscript line 156-159)

6. The example of "two-stage approach" is not convincing to support the "reference-free" ability of their model. In the manuscript, the authors have apriori knowledge of three T subtypes and two DC subtypes in this dataset, and they used these reference single cells to extract marker genes to process their model. However, it's important to note that reference-free models are usually considered in a situation where an ideal reference dataset is unavailable. In real-world data, we usually lack priori subtype information for a ST dataset or an idea reference dataset containing a hierarchical cell type annotation. Therefore, a one-stage deconvolution is more realistic for most real-world scenarios.

In Figure 3, for model testing and evaluation purposes, we used markers derived from the original data to minimize any confounding factors that may affect the performance of the two-stage model. For example, if an external set of markers was used and this resulted in low performance, it would be difficult to know whether this occurred because of a bad selection of marker genes or because of the model itself.

We do agree with the reviewer that when the two-stage model is used, the users should have some knowledge or make some assumptions about the subtypes contained in the cell type of interest. Conceptually, even in reference-based methods, users also make similar assumptions that their datasets (which may have different diseases, and biological and technical conditions) contain the same cell types as the cell types available in the reference dataset. Moreover, with the ability to include one or more “no-marker” cell types, SMART can discover novel cell types that have not been specified with marker genes. This is an advantage over the reference-based methods because the reference-based methods are restricted to the cell types available in the reference dataset.

Compared to a one-stage model, an advantage of the two-stage model is to re-use the marker genes to distinguish cell subtypes. For example, the proliferation marker gene MKI67 may be used for cell type A to distinguish proliferating cell type A from baseline cell type A and then re-used for cell type B to distinguish proliferating cell type B from baseline cell type B. A one-stage approach, however, may not correctly identify these subtypes because of redundant markers.

To validate the two-stage model in a real-world ST dataset, we extended the application of SMART on the 10X Visium mouse brain dataset (Figure 5) to a two-stage model. In this case, we assumed that the “neurons” have two subtypes: “excitatory neurons” and “inhibitory neurons”. We included a “no-marker” cell type in both stages, representing any cell types that were not specified in our marker gene list. The marker genes for excitatory neurons and inhibitory neurons were collected from the CellMarker 2.0 database. We showed in Figure 5B and 5C that the two-stage approach identified excitatory neurons and inhibitory neurons and their locations in the brain tissue were consistent with data from the literature: cortical layer 1 (the outer layer) contains mainly inhibitory neurons and excitatory neurons are much more abundant than inhibitory neurons. The “no-marker” cell type from stage one might correspond to a novel cell type in the medial habenula region while the proportion of the “no-marker” cell type from stage two was minimal and could be ignored. (See revised manuscript line 253-275)

7. In terms of SMART, it can also predict cell type-specific gene expression as part of the output. However, I did not find any assessment of the performance of this aspect. The authors should also evaluate the accuracy of gene expression prediction results. Particularly, assessing its performance in predicting cell counts in genome-wide data is important for users.

We thank the reviewer for the suggestion. We have now added Supplementary Figure 1 showing the top 10 highly expressed genes (ranked based on the relative gene count frequency) of the predicted cell type-specific gene expression in the MK dataset. We also added Figure 2F to compare the rank of the genes in the ground truth cell type-specific gene expression and those in the predicted cell type-specific gene expression. Each dot in Figure 2F represents a gene. We only included the gene if the relative predicted cell type-specific gene expression is >0.1% because the lowly expressed genes may be just background noise. We showed in Figure 2F that there is a high correlation between the gene rank in the ground truth and the predicted gene expression for each cell type. The ground truth highly expressed genes were also the predicted highly expressed genes. (line 112-115)

As mentioned in comment 4, we recommend including a gene selection procedure and only applying SMART on the marker genes and highly variable genes to avoid a long runtime. Therefore, although there were only ~300 genes in the main benchmarking analysis, it can still provide a good depiction of the performance of SMART because even for a genome-wide ST dataset, only a small subset of the genes will be used. We have also validated SMART on a genome-wide dataset in Figure 5 where we apply SMART to the 10X Visium mouse brain dataset. (line 253-275)

8. Figure 1 should be improved. The workflow within the SMART model box is too simple and will cause confusion to readers. The authors should emphasize the key components of the model intuitively. In addition, the authors should provide the annotations for these alphabet labels.

We thank the reviewer for the suggestion. We have now included the annotations for the alphabet labels. We have also added a summary of the model in the figure caption.

9. Since the heatmaps in Figure 2C, 3C and 4C show the Pearson correlation coefficients between the predicted and the GT cell type proportions for each cell type, why are these matrices asymmetrical?

These heatmaps show the correlation between the predicted cell type proportion (columns) and the ground truth cell type proportion (rows). Therefore, they do not have to be symmetrical because the rows and columns are different. For example, in Figure 2E (original Figure 2C), the correlation between the predicted proportions of “Podocyte” and the ground truth proportions of “LoHEpi” could be different from the predicted proportions of “LoHEpi” and the ground truth proportions of “Podocyte”.

10. Figure 4 line 4: "(E)" should be "(D)".

The figure caption has now been corrected.

Reviewer #2: Authors propose a topic model called SMART, which is closely based off the existing algorithm called keyATM to deconvolve spot from ST data. The overall writing is clear but the paper lacks technical contribution and misses some important intuition and analyses.

Major comments:
1. One major limitation of this approach is that it does not make use of the spatial information, which makes it a similar approach as other non-ST deconvolution methods.

We thank the reviewer for the comment. Similar to other unsupervised or semi-supervised topic model-based ST deconvolution methods such as STdeconvolve and Celloscope, although SMART does not use spatial information, it performs best on ST datasets. It best suits the situation where the proportional distribution of cell types across spots is heterogeneous and sparse (i.e., some spots have more cell-type A, and other spots have more cell-type B, etc.). This condition is generally met in ST data. In bulk transcriptomics data, however, the proportional distribution is more homogeneous across samples with much lower sparsity (which is equivalent to having a corpus of very long documents with similar topic distribution), preventing the model from making accurate estimates. Also, SMART uses inverse gene frequency to help improve the prediction accuracy. In the case of bulk transcriptomics, the inverse gene frequency becomes less relevant compared to ST data. As a semi-supervised method without a reference dataset, SMART also works best when there are a sufficiently large number of samples/spots/documents. This is also generally satisfied in ST data compared to bulk transcriptomics (more ST spots than bulk transcriptomics samples). We have added a section to the supplementary methods about the use of SMART on bulk transcriptomics data.

2. Since the proposed model does not use spatial info, how does it compare with well-known and recently developed non-spatial deconvolution method like Cibersort, MuSiC, BayesPrism, and GTM-decon. Latter two are reference-based guided topic model and able to deconvolve bulk transcriptome including ST spots.

As mentioned in comment 1, SMART works best on spots with heterogeneous cell type distribution. The ST spots contain much fewer cells (high sparsity) and are more heterogeneous in cell type compositions compared to bulk transcriptomics samples. For example, the 10X Visium platform contains only 1-10 cells on average while bulk RNA-seq samples may contain hundreds of thousands of cells. Also, the ST data are usually larger in sample size compared to bulk transcriptomics data (more ST spots than bulk transcriptomics samples). Additionally, since bulk transcriptomics data contain much more gene counts (lower sparsity) compared to spatial transcriptomics data, scalability could be another issue for applying LDA-based models on bulk transcriptomics data. SMART was developed and tuned specifically for spatial transcriptomics data with a small number of cells per spot, and we would not recommend applying SMART to bulk transcriptomics data.

To compare the performance of SMART against some of the bulk transcriptomics deconvolution methods, in addition to the benchmarking analysis in Figure 2, we also applied Cibersort, MuSiC and BayesPrism on the simulated MK ST dataset (Supplementary Figure 6). Since Cibersort, MuSiC, and BayesPrism are reference-based methods, the batch effect between the reference dataset and the ST dataset needs to be considered. Therefore, we performed the evaluation in a more realistic setting using an external dataset (the TMS dataset) as the reference for Cibersort, MuSiC, and BayesPrism; marker genes derived from the TMS dataset were used for SMART. As expected, SMART outperforms Cibersort, MuSiC, and BayesPrism with a smaller per-spot RMSE, indicating that SMART is more suitable for ST datasets than the bulk transcriptomics deconvolution methods. The bulk transcriptomics deconvolution methods are more specialized for bulk transcriptomics data and may not provide accurate estimates for ST datasets.

3. The authors wrote "if the variable equals 0, _di is drawn from the gene frequencies for cell types without marker genes _k". This makes no sense. If phi_k is ONE topic distribution over all genes except marker genes for cell type k, how can it be "the gene frequencies for cell types without marker genes" (note here cell types are plural)?

We thank the reviewer for pointing this out. $\phi_{k}$ should be the topic distribution (gene frequencies) over all genes for cell type $k$ (not plural). It has now been corrected in the main text.

4. In NLP, s_di=1 means that the keyword w_di is sampled from the key topic k whose distribution is over the keywords; s_di=0 means that the keyword is sampled from a standard topic, whose distribution is over the entire vocabulary. When s_di = 0, does that mean the cell is not cell type k but somehow expresses the marker gene?

We apologize for the confusion. Suppose the vocabulary contains *V* unique words (genes). For a topic (cell type) $k$ with keywords (marker genes), when $s_{di}$=1, the word $w_{di}$(an mRNA molecule) is sampled from the topic distribution of keywords $\tilde{\phi}_{k}$. This is a probability vector of length *V* with zeros for non-keywords and positive values for the keywords. When $s_{di}$=0, the word $w_{di}$is sampled from the standard topic distribution $\phi_{k}$. This is a probability vector of length *V* with positive values for all words. The final topic distribution $\phi_{k}^{*}$ for topic $k$ is then based on a mixture of the two distributions $\phi_{k}$ and $\tilde{\phi}_{k}$, weighted by the Bernoulli variable $\pi_{k}$. This mixture structure will make the prior means for the keywords have a higher frequency than the non-keywords. In terms of deconvolution, when $s_{di}$=1, it means that the mRNA molecule $w_{di}$ is a copy of a marker gene in cell type *k* with probability based on $\tilde{\phi}_{k}$, and when $s_{di}$=0, it means that the mRNA molecule could be either a copy of a marker gene or a non-marker gene in cell type $k$ with probability based on $\phi_{k}$. The final gene frequencies $\phi_{k}^{*}$ is a mixture of $\phi_{k}$ and $\tilde{\phi}_{k}$, making the prior means of the marker genes higher than non-marker genes, and thus, resembles the inflated expression of marker genes in cell type $k$.

5. Although the algorithm closely follows keyATM, sufficient details should still be provided for the Gibbs sampling algorithm.

We thank the reviewer for the suggestion. We have now added a section in the supplementary method providing some details on the sampling algorithm. (Supplementary Methods line 103-136)

6. To see how well each cell type being deconvolved, authors should evaluate the correlation per cell type across cells rather than correlation across all cells and cell types.

We agree with the reviewer that it is important to evaluate the correlation per cell type instead of across all cell types. In Supplementary Figure 3A (original figure 2E and 2F), we only used the correlation across all cell types to demonstrate that semi-supervised methods (i.e. SMART) are more reproducible and show smaller variances compared to unsupervised methods (i.e. STdeconvolve), which is one of the advantages of semi-supervised models. The RMSE across the cells (Supplementary Figure 3B) also showed similar results. We did not separate the comparison by cell type because STdeconvolve did not generate results for all cell types as mentioned in the main text.

In Figure 2E, 3C, and 4C where we assess the performance of SMART, we have shown the correlation between the SMART-predicted cell type proportions and the ground truth cell type proportions for each cell type separately.

7. A main benefits of topic model is the interpretable topic distribution over genes. Yet no topic analysis was performed in the paper. For example, what are the top genes for phi_k and phi_tilde for each cell type k?

We thank the reviewer for the suggestion. We have now added Supplementary Figure 1 showing the top 10 highly expressed genes (ranked based on the relative gene count frequency) of the predicted cell type-specific gene expression ($\phi_{k}^{*})$ in the MK dataset. We also added Figure 2F to compare the rank of the highly expressed genes in the ground truth cell type-specific gene expression and those in the predicted cell type-specific gene expression. Each dot in Figure 2F represents a gene. We only included the gene if the relative predicted cell type-specific gene expression is >0.1% because the lowly expressed genes may be just background noise. We showed in Figure 2F that there is a high correlation between the gene rank in the ground truth and the predicted gene expression for each cell type. The ground truth highly expressed genes were also the predicted highly expressed genes. (line 112-115)

8. I agree that having overlapping marker genes between cell types can lead to poor performance of the keyATM algorithm. However, the proposed two-stage approach is ad hoc and feels arbitrary.

The two-stage approach is a simple extension of the base or the covariate model to turn it into a hierarchical-like approach. The motivation for the two-stage approach is to distinguish highly similar cell subtypes or cell states. Sometimes, the cell subtypes or cell states may just be different by one cell marker. For example, MKI67 is the only marker that differentiates proliferating basal epithelial cells from basal epithelial cells. A one-stage approach may not work well in this case due to redundant marker genes because almost all marker genes for these two cell types are identical. With a two-stage approach, we may first obtain the estimates for basal epithelial cells and further distinguish the two cell types with the MKI67 gene. We chose a two-stage approach instead of more stages mainly to avoid the propagation of errors at each stage.

To validate the two-stage model in a real-world ST dataset, we extended the application of SMART on the 10X Visium mouse brain dataset (Figure 5) to a two-stage model. In this case, we assumed that the “neurons” have two subtypes: “excitatory neurons” and “inhibitory neurons”. We included a “no-marker” cell type in both stages, representing any cell types that were not specified in our marker gene list. The marker genes for excitatory neurons and inhibitory neurons were collected from the CellMarker 2.0 database. We showed in Figure 5B and 5C that the two-stage approach identified excitatory neurons and inhibitory neurons and their locations in the brain tissue were consistent with data from the literature: cortical layer 1 (the outer layer) contains mainly inhibitory neurons and excitatory neurons are much more abundant than inhibitory neurons. The “no-marker” cell type from stage one might correspond to a novel cell type in the medial habenula region while the proportion of the “no-marker” cell type from stage two was minimal and could be ignored. (line 253-275)

Minor comments:
1. Fig. 2: Having the groundtruth composition on the side is helpful for comparison.

We have now added a pie chart for the ground truth composition to the main benchmarking analysis (Figure 2C)

2. Fig. 4 caption panel E should have been panel 'D'

The figure caption has now been corrected.

**Second round of review**

**Reviewer 1**

The manuscript has seen significant improvement and has effectively addressed the majority of the previous comments and concerns. However, there is still one concern regarding the authors’ response to my previous comment 1 that needs to be addressed before publication.

1. In Figure 2G, utilizing CellMarker genes results in a much lower RMSE compared to using TMS markers, highlighting the substantial impact of marker gene selection strategy on SMART’s accuracy. The authors may consider dedicating another paragraph to discuss marker gene selection to provide further guidance to users. For instance, they could compare the differences in gene markers between the GT, CellMarker, and TMS marker gene sets using a Venn plot. Additionally, a table could be utilized to illustrate how shared or unique genes in each set contribute to the SMART predictions, thus examining how SMART’s performance varies with different sets. If SMART maintains high performance despite limited shared genes, it would demonstrate its high robustness.

**Reviewer 2**

I would like to thank authors for the efforts in addressing my comments. However, some of the major comments were either not fully addressed or require further efforts:
1. The authors do not answer my comment 1 about not using spatial information. I was asking why spatial information such as the relative distance between spots and coordinates is not used, the authors instead gave me an explanation about the distribution difference between ST and bulk data.
2. The comparison is not sufficient. Supp. Fig. 6 shows only the overall deconvolution performance. And only RMSE is shown. As in my comment 6, it is important to see how well each cell type being deconvolved, i.e., RMSE AND correlation per cell type across cells. Basically, authors should show the performance using the same way as in the new Supp. Figure 3.
3. The authors should also compare their method with GTM-decon, which was shown to perform competitively against BayesPrism and Cibersortx.
4. For my comment 6, I understand that STdeconvolve does not generate identifiable topics but the topics can be identified with specific cell types in the post-hoc way. The diagonal values in Fig. 2C can be show separately for each cell type as barplot with error bar indicating standard deviation over repeated runs (e.g., simulation runs). This comment is also related to my Comment 2 in the additional method comparison.
5. For Supp. Figure 1, can authors label the known marker genes among the top 10 genes for the corresponding cell types? Is there statistically significant enrichments?

Minor comments:
- Typo in all places: "Categorial" => "Categorical"

**Authors Response**

**Point-by-point responses to the reviewers’ comments:**

Reviewer reports:

Reviewer #1: The manuscript has seen significant improvement and has effectively addressed the majority of the previous comments and concerns. However, there is still one concern regarding the authors’ response to my previous comment 1 that needs to be addressed before publication.

1. In Figure 2G, utilizing CellMarker genes results in a much lower RMSE compared to using TMS markers, highlighting the substantial impact of marker gene selection strategy on SMART’s accuracy. The authors may consider dedicating another paragraph to discuss marker gene selection to provide further guidance to users. For instance, they could compare the differences in gene markers between the GT, CellMarker, and TMS marker gene sets using a Venn plot. Additionally, a table could be utilized to illustrate how shared or unique genes in each set contribute to the SMART predictions, thus examining how SMART’s performance varies with different sets. If SMART maintains high performance despite limited shared genes, it would demonstrate its high robustness.

We thank the reviewer for the suggestion. We added Supplementary Figure 5 to show the overlap of the three marker gene sets (GT, TMS, and literature-based marker genes) for each cell type. Additionally, we have added Supplementary Table 4 to show the top 10 SMART-predicted cell type-specific genes when each set of marker genes was used. Each gene is labeled as “Y” if used as a marker gene. The shared marker genes (with at least one other set) are labeled with an “s”.

As expected, most of the top 10 predicted genes for each set were also the input marker genes. These marker genes contained both genes shared between the marker gene sets and unique marker genes in each set. Thus, these data indicate that different marker genes can be used with SMART, although the shared marker genes, which are considered more reliable, probably impose a greater impact on the overall results. Notably, the top 10 genes also contained non-marker genes, indicating the robustness of SMART to the inclusion of marker genes that were of reduced quality or missing altogether from the input, so long as the overall proportion of such genes is relatively small. We therefore recommend that the users consult multiple sources to identify and select the most reliable marker genes (i.e., marker genes that have been confirmed by multiple sources). SMART provides robust estimates with as few as three marker genes, assuming good quality of the marker genes. Ideally, however, having, on average, 10 marker genes per cell type significantly improves the quality of the results (Supp Figure 9). We have added lines 291-300 and 352-356 in the main text to provide a deeper insight into the marker selection process.

Reviewer #2: I would like to thank authors for the efforts in addressing my comments. However, some of the major comments were either not fully addressed or require further efforts:
1. The authors do not answer my comment 1 about not using spatial information. I was asking why spatial information such as the relative distance between spots and coordinates is not used, the authors instead gave me an explanation about the distribution difference between ST and bulk data.

We thank the reviewer for this comment. In our study, we did try to incorporate spatial information in our analyses. One way we tried is as follows: we first calculated the neighboring information of the spots and clustered them based on both their spatial location and gene expression. After this, the spatial clusters were used as a covariate in a SMART-covariate model. However, compared to the baseline model, which did not use spatial information, the performance of the spatially incorporated SMART-covariate model was not significantly improved (P>0.05) using the MK dataset, as shown in the following figure. Therefore, for parsimony, we chose not to include spatial information in SMART. Further, in the paper, we showed that the baseline SMART model that does not include spatial information demonstrated superior performance to some of the best-performing methods that include spatial information (i.e., cell2location, CARD).

2. The comparison is not sufficient. Supp. Fig. 6 shows only the overall deconvolution performance. And only RMSE is shown. As in my comment 6, it is important to see how well each cell type being deconvolved, i.e., RMSE AND correlation per cell type across cells. Basically, authors should show the performance using the same way as in the new Supp. Figure 3.

We thank the reviewer for the suggestion. We have modified the figure (now Supp. Fig. 11) to include the RMSE and correlation across all spots and in each cell type. SMART demonstrated the best performance compared to the bulk deconvolution methods for all cell types.

3. The authors should also compare their method with GTM-decon, which was shown to perform competitively against BayesPrism and Cibersortx.

We thank the reviewer for these comments. In the revised text, we added DWLS, SCDC, and Bisque, which have been shown to be some of the best-performing bulk deconvolution methods according to recent benchmarking studies (Avila Cobos *et al.*, *Nat Commun*, 2020; Huuki-Myers *et al.*, *bioRxiv*, 2024). The Supplementary Methods and Supp Figure 11 have been updated with these three methods added. As for GTM-decon, we could not obtain results from it because of unsolvable errors in the software package. To resolve the problem, we tried to contact the authors but did not receive a response.

4. For my comment 6, I understand that STdeconvolve does not generate identifiable topics but the topics can be identified with specific cell types in the post-hoc way. The diagonal values in Fig. 2C can be show separately for each cell type as barplot with error bar indicating standard deviation over repeated runs (e.g., simulation runs). This comment is also related to my Comment 2 in the additional method comparison.

In addition to Supp Figure 11, which addresses comment 2, we have also added:

1) Supp Figure 2, which shows the correlation and RMSE across all spots for the main benchmarking analysis using the MK dataset. The error bars were not included in this figure because most methods included in the comparison do not change over repeated runs. For SMART, STdeconvolve, and Celloscope, which do change over repeated runs, they were further compared in Supp Figure 8, which includes error bars indicating standard deviation over repeated runs.
2) Supp Figures 3 and 4, which show the correlation and RMSE in each cell type for the main benchmarking analysis. Supp Figure 3A includes the diagonal values shown in Fig. 2C. Similarly, the error bars were not included in this figure but in Supp Figure 8.
3) Supp Figure 8, which shows the correlation and RMSE over 100 repeated runs in each cell type for SMART, STdeconvolve, and Celloscope. The error bars indicate the standard deviation over repeated runs.

The latent cell types identified by STdeconvolve and CARDfree were annotated using gene set enrichment analysis (lines 497-506 in the main text). As mentioned in lines 175-180 in the main text, when gene set enrichment analysis was used to annotate the latent cell types, STdeconvolve and CARDfree results rarely contained all eight cell types. Therefore, although we did 100 repeated runs for STdeconvolve, the number of repeated runs identifying each cell type varies and is labeled in Supp Figure 8. If not the best, SMART was one of the best methods for most cell types in all three benchmarking scenarios.

5. For Supp. Figure 1, can authors label the known marker genes among the top 10 genes for the corresponding cell types? Is there statistically significant enrichments?

We have now labeled the known marker genes among the top 10 genes with checkmarks in Supp Figure 1. The marker genes were significantly enriched in all cell types (Fisher’s exact test P<0.001).

Minor comments:
- Typo in all places: "Categorial" => "Categorical"

We have corrected the typo in all places.

**Third round of review**

**Reviewer 1**

The authors have addressed my concerns in this revision.

**Reviewer 2**

All my comments were addressed except a major one:

As SMART-covariate model does not improve the deconvolution when spatial info is used, it presents somewhat minimal technological advances given the recently published work such as GTM-decon, which is also a guided topic model. Therefore, the authors will need to compare their approach with GTM-decon.

Authors said that they encountered error in running the software and asked the GTM-decon corresponding author and received no reply. Sorry for being meticulous here but what error did the authors experience? They should email the GTM-decon corresponding author (Yue Li; [yueli@cs.mcgill.ca](mailto:yueli@cs.mcgill.ca)).

**Authors Response**

**Point-by-point responses to the reviewers’ comments:**

Reviewer reports:

Reviewer #1: The authors have addressed my concerns in this revision.

Reviewer #2: All my comments were addressed except a major one:

As SMART-covariate model does not improve the deconvolution when spatial info is used, it presents somewhat minimal technological advances given the recently published work such as GTM-decon, which is also a guided topic model. Therefore, the authors will need to compare their approach with GTM-decon.

Authors said that they encountered error in running the software and asked the GTM-decon corresponding author and received no reply. Sorry for being meticulous here but what error did the authors experience? They should email the GTM-decon corresponding author (Yue Li; yueli@cs.mcgill.ca).

We apologize for not including GTM-decon in our previous submission. We have received a reply from the authors of GTM-decon a few weeks ago. The error we experienced was due to an incompatible version of its dependency library Armadillo. Following the author’s suggestion, we recompiled the source files with the correct version of Armadillo, and the issue has now been resolved.
We have added GTM-decon to the bulk deconvolution methods comparison (Supplementary Methods line 155-169, Supp Figure 11). SMART demonstrated improved performance over GTM-decon with a stronger correlation and a lower RMSE. This may be because GTM-decon is more specialized for bulk RNA-seq deconvolution. A scRNA-seq reference profile is also needed in GTM-decon to obtain the priors. Moreover, in GTM-decon, the bulk RNA-seq data must be sparsified to identify novel cell types with regular LDA, which may cause information loss. On the other hand, SMART, as a marker gene-assisted method, uses two separate Dirichlet distributions for marker genes and non-marker genes, and does not need a scRNA-seq reference profile. It also performs better than GTM-decon on spatial transcriptomics data.
